# Activation of *KIT* signaling promotes early tumorigenesis through the AP-1 pathway in *APC/TP53* double-knockout human colon organoids

**DOI:** 10.1038/s41419-026-09056-7

**Published:** 2026-07-04

**Authors:** Younghee Choi, Eunju Kim, Soo-Yeon Cho, Jihyun Park, Youngwon Cho, Sungho Kim, Sang-Hyun Song, Tae-You Kim

**Affiliations:** 1 Department of Molecular Medicine and Biopharmaceutical Sciences, Graduate School of Convergence Science and Technology, Seoul National University, Seoul, Republic of Korea; 2 Cancer Genomics Research Laboratory, Cancer Research Institute, Seoul National University, Seoul, Republic of Korea; 3 State University of New York Upstate Medical University, Syracuse, NY USA; 4 Department of Internal Medicine, Seoul National University Hospital, Seoul, Republic of Korea

**Keywords:** Colon cancer, Chromatin remodelling

## Abstract

Colorectal cancer (CRC) develops through a series of progressive genetic mutations, with alterations in *APC* and *TP53* being the most frequently observed in the early stages. Although the loss of *APC* is recognized as a significant initiating event, the epigenetic processes through which the simultaneous inactivation of *APC* and *TP53* facilitates the onset of colorectal tumorigenesis remain poorly characterized. To address this gap, we developed a human colon organoid model using CRISPR-Cas9 to achieve a double knockout of *APC* and *TP53*. Through extensive multi-omics profiling, we characterized the epigenetic landscape distinctive of early-stage CRC. We identified *KIT*, a receptor tyrosine kinase, as a critical oncogenic driver that is significantly upregulated in ΔDKO (*APC* and *TP53* double knockout) organoids, thereby activating the MAPK and Wnt signaling pathways to augment proliferation and tumorigenesis. Furthermore, AP-1 transcription factors (FOS/JUN) regulate *KIT* expression via chromatin remodeling. Functional analyses indicated that KIT is integral to sustaining the elevated proliferation rates observed in ΔDKO organoids. These findings reveal a novel AP-1/KIT signaling axis that is central to the early progression of CRC, thereby presenting a promising avenue for therapeutic intervention.

**Figure:**
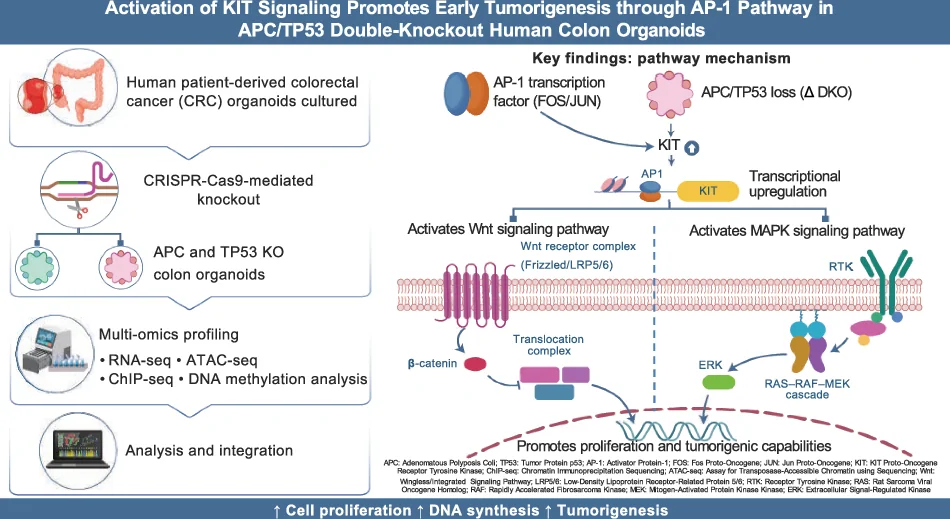

## Introduction

Colorectal cancer (CRC) progresses through a well-characterized adenoma-to-carcinoma sequence driven by the accumulation of distinct genetic alterations [1, 2]. Among these, loss-of-function mutations in *APC* are observed in over 80% of human CRCs, widely recognized as an initiating event in tumorigenesis through constitutive activation of the Wnt signaling pathway and the promotion of benign polyp formation [3, 4]. Furthermore, mutations in the *TP53* gene are present in over 50% of CRC cases, contributing to tumor advancement by inducing genomic instability and facilitating the evasion of apoptosis [5–7]. Beyond its conventional functions, *TP53* also serves as an epigenetic regulator that maintains chromatin integrity by modulating DNA methylation and histone modifications [7]. Although co-mutation of *APC* and *TP53* is frequently documented in CRC, the epigenetic mechanisms underpinning their synergistic involvement in the early stages of tumorigenesis remain poorly elucidated, partly due to the lack of physiologically relevant human model systems that accurately replicate early-stage disease.

Organoid technology has emerged as a revolutionary platform in oncology research, overcoming the limitations of traditional transgenic murine models and immortalized cell lines by providing a physiologically relevant human model system [8, 9]. Human colon organoids effectively replicate the fundamental genetic and functional characteristics of the originating tissue, thereby facilitating high-resolution modeling of the molecular mechanisms that govern early neoplastic transformation [10]. This system also presents a distinctive opportunity to investigate epigenetic regulation within a native-like chromatin milieu that reflects the in vivo tumor microenvironment [11, 12].

Epigenetic modifications, such as alterations in chromatin accessibility and histone modifications, are increasingly recognized as critical determinants of transcriptional reprogramming in the context of cancer [13–15]. Recent studies have leveraged histone mark-based chromatin state profiling in CRC organoids to identify key transcription factors that drive enhancer activity [16], and large-scale pan-cancer epigenomic atlases have laid the foundation for therapeutic target discovery [17]. However, how early genetic mutations reshape the chromatin landscape to establish tumor-permissive transcriptional programs remains unclear in the context of CRC.

To bridge this gap, we developed a CRISPR-Cas9–engineered human colon organoid model featuring *APC* and *TP53* double knockout (ΔDKO), which serves as a genetically and physiologically relevant system for examining early-stage CRC. Using this model, we conducted integrated multi-omics profiling, including RNA-seq, ATAC-seq, ChIP-seq, and DNA methylation analyses, to unravel the dynamic evolution of the epigenetic landscape during neoplastic transformation. Through clustering and motif analysis, we identified the AP-1 transcription factor complex (FOS/JUN) as a key epigenetic regulator that was upregulated following co-inactivation of *APC* and *TP53*. The AP-1 complex directly facilitates chromatin remodeling and transcriptional activation of the receptor tyrosine kinase *KIT*, thereby enhancing the MAPK and Wnt signaling pathways to sustain hyperproliferation. Genetic and pharmacological perturbations of the AP-1/KIT signaling axis inhibited oncogenic signaling and disrupted tumorigenic phenotypes in ΔDKO organoids, indicating that this pathway is a crucial driver of early colorectal cancer progression.

## Materials and methods

### Human specimens

Human colon tissues were obtained from the Seoul National University Hospital (Seoul, Korea). Following surgical resection, portions of each colorectal tumor and adjacent normal tissue were immediately snap-frozen and stored in liquid nitrogen until further use. The remaining tissue samples used to establish primary cultures underwent pathological diagnosis. All human tissue samples were obtained with informed consent from the participants or their legally authorized representatives, in accordance with protocols approved by the Institutional Review Board of Seoul National University Hospital (permission number: H1708-031-875). This study was conducted in accordance with the principles of the Declaration of Helsinki.

### Establishment of patient-derived organoids

Normal and tumor tissues were established and cultured following the protocol described by Cho et al. [18]. In short‚ normal mucosa (*n* = 3; #203 (Age 67, Female), #208 (Age 62, Male), #211 (Age 69, Male)) was cut into 1–2-mm pieces and washed with Dulbecco’s phosphate-buffered saline (DPBS) until the supernatant was clear to separate the crypts. The normal colonic mucosal fragments were then washed with 2 mm EDTA/DPBS chelation buffer and incubated on ice under shaking conditions. After 1 h‚ the crypts were scraped off the tubes and washed with basal medium‚ which was advanced DMEM/F12 (Invitrogen‚ Carlsbad‚ CA‚ USA) supplemented with penicillin/streptomycin (Invitrogen)‚ 10 mm‚ HEPES (Invitrogen), and GlutaMAX (Invitrogen).

For tumor organoids (*n* = 1; #203 (Age 67, Female)), tumor tissues were washed with DPBS and dissociated with a gentleMACS Dissociator (Miltenyi Biotec‚ Bergisch Gladbach‚ Germany) and run through a 70 μm cell strainer to remove debris. Cells were then resuspended in basal medium․ Normal crypts from the colon and dissociated tumor cell pellets were embedded in a solution of Matrigel (Corning‚ Corning‚ NY‚ USA) and plated on 24-well plates.

### Culture of patient-derived organoids

Organoid culture medium was added once the Matrigel had solidified. The organoid culture medium contained: 50% Wnt-3a or the hAFM/Wnt-3a conditioned medium (only for the normal organoids)‚ 10% R-spondin1 conditioned medium (only for the normal organoids)‚ 10% Noggin conditioned medium or 100 ng/mL recombinant Noggin (PeproTech‚ Cranbury‚ NJ‚ USA)‚ 50 ng/mL recombinant human epidermal growth factor (EGF) (PeproTech)‚ B27 (Invitrogen)‚ 1.25 mm N-acetyl cysteine (Sigma-Aldrich‚ St. Louis‚ MO‚ USA)‚ 10 mm nicotinamide (Sigma)‚ 3 μm SB202190 (Sigma)‚ 500 nm A83-01 (Tocris‚ Bristol‚ UK)‚ 10 nm prostaglandin E2 (Sigma)‚ 10 nm gastrin (Sigma)‚ and 100 μg/mL Primocin (InvivoGen‚ San Diego‚ CA‚ USA) in the basal medium. The Wnt-3a/Noggin conditioned medium cell lines were kindly provided by H. Clevers‚ and the hAFM/Wnt-3a conditioned medium cell line was kindly provided by Prof. Junichi Takagi [19]. The R-spondin1 conditioned medium cell line was purchased from Trevigen (Gaithersburg‚ MD‚ USA)․

### Organoid culture and passaging

Organoids were embedded in Matrigel for culturing, and the medium was replaced three times per week. During passaging, both organoids and Matrigel were harvested, and the Matrigel was removed with cold DPBS. Organoids were then fragmented into clusters of approximately 2–10 cells using Accutase (Stem Cell Technologies, Canada) and replated in fresh Matrigel. Only organoids below passage 10 were used in all the experiments. For experimental procedures, samples were collected on day 6 post-seeding, with the media refreshed on days 2 and 5. Organoids were regularly tested for mycoplasma contamination.

### Genomic engineering: CRISPR-Cas9 mediated knock out and overexpression

For generating sequential knockout of genes, normal human colon organoids were transduced by using the lentiCRISPRv2 plasmids (Addgene; plasmids #52961) carrying sgRNA targeting the *APC* and a puromycin resistance․The transduced cells were then selected by 1 μg/mL puromycin (Sigma-Aldrich; P8833) for 2 weeks. Individual organoids from this positively selected population were then isolated and expanded into a number of single cell-derived *APC* knockout (Δ*APC*) clones, with successful biallelic inactivation of *APC* verified by genotyping and Western blotting․

To target *TP53*, the previously transduced Δ*APC* clones were transduced with a second lentiCRISPRv2 vector (Addgene; plasmids #83480) that contained an sgRNA against *TP53* and a blasticidin resistance. 1 μg/mL blasticidin (Sigma-Aldrich; SBR00022) was added to select positively transduced cells, and the culture was continued for 2 weeks. Single-cell-derived organoid clones were picked and expanded, confirming *TP53* knockout by genotyping and Western blotting, to generate double knockout (ΔDKO) organoids․

For *KIT*, *FOS*, and *JUN* knockout, ΔDKO organoids were transduced with lentiCRISPRv2 plasmids (Addgene; plasmids #98291) carrying specific sgRNAs of the respective genes. Following transduction, cells were subjected to selection with 1.5 μg/mL hygromycin (Invitrogen #10687010) for approximately two weeks. *KIT*, *FOS*, and *JUN* knockouts were maintained as bulk-selected populations. All sgRNA sequences used in this study are listed in Table 1. To reduce potential off-target effects, sgRNAs were selected using multiple independent design platforms (CRISPOR, CHOPCHOP, and RGEN), and guides consistently ranked among the top candidates across these tools were preferentially used.

**Table 1:** Oligo sequences for sgRNA used in CRISPR knockout.

	Sequences (5'→ 3')
APC_sgRNA_sense	CACCGGTTTGAGCTGTTTGAGGAGG
APC_sgRNA_antisense	AAACCCTCCTCAAACAGCTCAAACC
TP53_sgRNA_sense	CACCGGAGCGCTGCTCAGATAGCGA
TP53_sgRNA_antisense	AAACTCGCTATCTGAGCAGCGCTCC
KIT_sgRNA_sense	CACCGACCGCGATGAGAGGCGCTCG
KIT_sgRNA_antisense	AAACCGAGCGCCTCTCATCGCGGTC
FOS_sgRNA_sense	CACCGAACCGCCACGATGATGTTCT
FOS_sgRNA_antisense	AAACAGAACATCATCGTGGCGGTTC
Jun_sgRNA_sense	CACCGGATTATCAGGCGCTCCAGCT
Jun_sgRNA_antisense	AAACAGCTGGAGCGCCTGATAATCC

To overexpress *KIT*, the pCMV3-ORF (#HG11996-UT) vector was introduced into ΔGFP organoids. KIT overexpression was confirmed by western blot analysis one week after transfection.

For viral production, lentiviral vectors were introduced into 293FT cells using the Virapower packaging mix (Invitrogen, K4975-00), Opti-MEM (Gibco, Baltimore, MD, USA), and Lipofectamine 2000 (Invitrogen, 52887). The viral supernatants were collected after 2 days. Organoids dissociated into single cells were exposed to the viral supernatant for 4 h in the presence of 6 μg/mL polybrene (Sigma-Aldrich, H9268), 10 μM Y-27632 (Selleckchem, S1049), and 5 μM CHIR-99021 (Selleckchem, S2924) on ultra-low attachment plates (Corning), which were periodically agitated. Following infection, cells were re-embedded in Matrigel.

### Genomic DNA PCR and TA cloning

Genomic DNA was extracted from organoids using a QIAamp DNA Mini kit (Qiagen, Hilden, Germany). PCR amplification of the extracted DNA was conducted using Taq polymerase premix (Takara, #R004, Tokyo, Japan). The resulting PCR products were cloned into the T&A vector using a T&A Cloning kit (PBC Bioscience, Taipei, Taiwan). Sanger sequencing was performed using the M13 forward primer to verify the genomic DNA sequences. The primers used for the genomic PCR are listed in Table 2.

**Table 2:** Primer sequences used in genomic DNA PCR.

	Sequences (5'→ 3')
APC-gDNA-For	CCCTAGAACCAAATCCAGCA
APC-gDNA-Rev	CACTCAGGCTGGATGAACAA
TP53-gDNA-For	CCATGGGACTGACTTTCTGC
TP53-gDNA-Rev	GTTTCCGTCTGGGCTTCTTG
KIT-gDNA-For	CCGGCATTAACACGTCGAAA
KIT-gDNA-Rev	CAAAAGCCACCCCAAACTCG

### Organoid viability assay (ATP luciferase assay)

20,000 organoids per well were plated in a 48-well plate and cultured. ATP quantification of organoids was performed on day 6 post-seeding. ATP levels were measured using an ATP assay kit (Promega #G9682, CellTiter-Glo) according to the manufacturer’s instructions. An equal volume of ATP detection reagent was added to the medium in each well, and the mixture was incubated at room temperature for 20 min. After incubation, 200 μL of the mixture was transferred to a 96-well white plate, and luciferase activity was measured.

### Immunohistochemistry (IHC) and H-score assessment

For IHC, organoids were harvested with Organoid Harvesting Solution (Trevigen, Gaithersburg, MD, USA) and fixed in 10% formalin. Paraffin-embedded organoids were sectioned at 4 μm, dried at 60 °C for 1 h, and deparaffinized using Ventana EZprep. Antigen retrieval was done with CC1 (Ventana) at 100 °C. After peroxidase blocking (OptiView), slides were incubated with primary antibodies against Ki67 (1:300, Invitrogen) and CK20 (1:100, Abcam) for 24 min at 37 °C. Detection used the OptiView HQ Universal Linker and DAB, followed by hematoxylin counterstaining and bluing. Slides were dehydrated and mounted (CS703).

Images were scanned (Aperio AT2), and the Ki-67 index was quantified as the percentage of nuclei with positive staining based on optical density.

$${Ki}-67\,{index}=({Ki}-67\,{positive}\,{cells}\,/\,{total}\,{counted}\,{cells})\,\times\,100$$

The maximum possible Ki-67 index was 100, while the minimum was 0.

### EdU incorporation assay

For the EdU assay, organoids were collected using organoid harvesting solution (Trevigen) and were fixed in 10% formalin at room temperature. The organoids were fixed and permeabilized with formaldehyde and Triton X-100. The EdU incorporation assay was conducted using the Click-iT Plus EdU Alexa Fluor 594 Imaging kit (Life Technologies), and EdU (10 µM) was added to Matrigel-embedded organoids and incubated for 4 h. The EdU reaction cocktail was added according to the manufacturer’s instructions. After 30 min of incubation, the DNA was stained and detected.

### Tissue genomic DNA extraction, library construction, and sequencing

Genomic DNA was isolated from adjacent normal tissue and tumor tissue from patient #203 using the QIAGEN DNeasy Blood & Tissue Kit according to the manufacturer’s instructions. Whole-exome sequencing was performed on both samples, with average coverages of 100× for the adjacent normal tissue and 300× for the tumor tissue.

The quality and‌ quantity of genomic DNA were assessed‌ using PicoGreen and agarose gel electrophoresis. For fragmentation, 0.5 μg of genomic DNA was diluted in EB buffer and subjected to sonication using a Covaris LE220 focused ultrasonicator (Covaris, Woburn, MA, USA) according to the manufacturer’s protocol‌ to obtain an average DNA fragment‌ size of 150 to 200 bp. The exomes were‌ prepared using the Agilent SureSelectXT Low Input Target Enrichment‌ protocol for the Illumina paired-end sequencing platform. The fragments were end-repaired,‌ A-tailed and‌ ligated to Agilent adapters before PCR amplification and purification․ The quality of the library and the distribution of the size were evaluated‌ using TapeStation DNA‌ ScreenTape D1000 (Agilent)․

For exome capture, 250 ng DNA library was hybridized to a SureSelect All Exon capture library (Agilent) and captured libraries were washed, increased,‌ and purified following the Agilent‌ SureSelect Target Enrichment protocol. The‌ final libraries were quantified by‌ qPCR using KAPA Library Quantification Kit for Illumina (Kapa Biosystems) sequencing platforms and qualified using TapeStation DNA ScreenTape D1000 system (Agilent)․

It was sequenced on the Illumina platform using sequencing-by-synthesis chemistry, and the output images were processed with the Illumina Real-Time Analysis (RTA) software for initial processing. The resulting binary base call files (․bcl) were then converted to fastq format with the Illumina bcl2fastq‌ v2․20․0 software, where the --barcode-mismatches parameter was set to 0․

### Bioinformatic analysis of whole-exome sequencing

The maftools R package (version 2.22.0) in the R environment (version 4.5.3) was used to visualize the somatic mutational landscape of the #203 tumor tissue. Somatic variants were converted to a standardized file format for reporting somatic variants named Mutation Annotation Format (MAF) containing gene symbols‚ genomic coordinates‚ reference/tumor alleles‚ and variant classifications. An oncoplot was then produced to visualize *APC* and *TP53* genes. For the final figure‚ unnecessary elements from the initial oncoplot‚ such as the TMB stack bar and the percent of variants for each gene‚ were removed‚ and all of the different classifications were colored using the maftools’ default color palette․

### RNA extraction, RT-qPCR, and RNA-sequencing

Total RNA was isolated using the TRI reagent (Molecular Research Center, Cincinnati, OH, USA; TR-118) in accordance with the manufacturer’s protocol. Two micrograms of RNA were reverse transcribed to synthesize cDNA, and RT-qPCR analysis was carried out as previously described [20]. Primer sequences used for RT-qPCR are listed in Table 3. Sequencing libraries were prepared according to the standard Illumina protocol for high-throughput sequencing. The transcriptome was sequenced on a Genome Analyzer IIx (Illumina), as described previously [21].

**Table 3:** Primer sequences used in RT-qPCR.

Gene	Sequences (5'→3')
18s_mRNA_F	AAACGGCTACCACATCCAAG
18s_mRNA_R	CCTCCAATGGATCCTCGTTA
AXIN2_mRNA_F	CCTGCCACCAAGACCTACAT
AXIN2_mRNA_R	CTTCATTCAAGGTGGGGAGA
LGR5_mRNA_F	GGAAATCATGCCTTACAGACC
LGR5_mRNA_R	CACTCCAAATGCACAGCACTG
MYC_mRNA_F	GCGACTCTGAGGAGGAACAA
MYC_mRNA_R	CCTCCAGCAGAAGGTGATCC
CCND1_mRNA_F	GGTCTGCGAGGAACAGAAGT
CCND1_mRNA_R	GAAATCGTGCGGGGTCATTG
LEF1_mRNA_F	GCAGACTGGTTTGCAGTGAA
LEF1_mRNA_R	ATGACAGTTTTGGGCAAAGG
BAX_mRNA_F	TTCATCCAGGATCGAGCAGG
BAX_mRNA_R	TGAGACACTCGCTCAGCTTC
ID1_mRNA_F	GGCTCCGCACTCTCATTC
ID1_mRNA_R	TGAAACAGAATGGGCAAAGC
CDKN1A_mRNA_F	ATGAAATTCACCCCCTTTCC
CDKN1A_mRNA_R	CCCTAGGCTGTGCTCACTTC
KIT_mRNA_F	TCTGACGTCAATGCTGCCAT
KIT_mRNA_R	GGCAGTACAGAAGCAGAGCA
EGR1_mRNA_F	TGACCGCAGAGTCTTTTCCT
EGR1_mRNA_R	TGGGTTGGTCATGCTCACTA
BCL3_mRNA_F	TCTTCGGGACCAGGATTTGC
BCL3_mRNA_R	GGGAGGGGACATGGAAATGG
VEGFD_mRNA_F	CCAAGTTCCCCATCCCTGTC
VEGFD_mRNA_R	GTCCCCTCCTCTCCATTCCT
MMP1_mRNA_F	CCTGAAGAATGATGGGAGGCA
MMP1_mRNA_R	CTCTTGGCAAATCTGGCGTG
CD44_mRNA_F	GCAATGCTTCTCAGACCACA
CD44_mRNA_R	GAGGGGAGAGGGTAGACAGG
CA2_mRNA_F	AAGGAACCCATCAGCGTCAG
CA2_mRNA_R	ACAAAGCAACCAGGGTCCAA

### Bioinformatic analysis of RNA-sequencing

After paired-end reads were trimmed with Trimmomatic to remove adapter sequences and low-quality bases, they were mapped to the human hg19 reference genome using STAR with default parameters. The resulting mapped BAM files were converted into HOMER tag directories using makeTagDirectory for downstream analysis. Gene-level raw read counts were obtained using exon-based counting via the analyzeRepeats.pl script. Based on this count matrix, differential gene expression analysis was performed using the HOMER script getDiffExpression.pl. Differentially Expressed Genes (DEGs) were determined with the criterion of FDR < 0.05 and fold change (|FC | ) >1.5 or 2. To visualize RNA-seq signals across genomic loci, gene track files were generated with HOMER’s makeUCSCfile and visualized in genome browsers. All RNA-seq experiments were performed with biological replicates.

### Assay for transposase-accessible chromatin(ATAC)-sequencing library construction

Organoids were dissociated into single cells using Accutase, and 1 × 10⁵ cells were collected. An ATAC-seq kit (Diagenode) was used to generate an ATAC-seq library. Cells were lysed, followed by tagmentation and DNA purification, and library amplification according to the manufacturer’s protocol. ATAC libraries were sequenced using the NextSeq platform (Illumina Inc.).

### Bioinformatic analysis of ATAC-sequencing

Paired-end reads were trimmed using Trimmomatic to remove adapter sequences and low-quality bases. The trimmed reads were mapped to the human hg19 reference genome using Bowtie2 with default parameters. The aligned reads were filtered to remove mitochondrial reads and PCR duplicates using SAMtools and Picard. The processed BAM files were then converted into HOMER tag directories using makeTagDirectory, serving as a unified input format for downstream analyses. Accessible chromatin regions were identified by calling peaks using HOMER’s findPeaks tool with the -style factor option. After merging peaks from each replicate, differential accessibility analysis was performed using HOMER’s getDiffExpression.pl with a cutoff of |FC | > 2, and FDR < 0.05. Peak annotation and normalized peak intensity quantification were performed using HOMER’s annotatePeaks.pl, which calculates tag counts normalized by library size for each peak region. Transcription factor binding motifs enriched within peaks were identified using HOMER’s findMotifsGenome.pl with the -size given option. For visualization, tracks were generated using the makeUCSCfile tool and viewed in the IGV. All ATAC-seq experiments were performed with biological replicates.

### Chromatin immunoprecipitation (ChIP)-sequencing

On day 6 post-transduction, 2 × 10⁷ organoids were collected and crosslinked using 1% formaldehyde for 10 min at room temperature. The reaction was stopped by the addition of glycine at a final concentration of 0.125 M for 5 min. Organoids were lysed with 1 mL of cell lysis buffer (10 mM Tris-Cl (pH 8.0), 10 mM NaCl, and 0.2% NP-40) supplemented with protease inhibitors and incubated for 30 min at 4 °C. Nuclei were separated by centrifugation at 3000 rpm for 5 min, and the pellets were resuspended in 500 μL of nuclei lysis buffer (50 mM Tris-Cl (pH 8.0), 10 mM EDTA, and 1% SDS) containing protease inhibitors, followed by a 15-min incubation at 4°C. Chromatin was sheared by sonication using Misonix 3000 to achieve an average fragment size of 200–400 bp.

The sonicated chromatin was diluted with 2.5 mL of IP dilution buffer (20 mM Tris-Cl (pH 8.0), 150 mM NaCl, 2 mM EDTA, 0.01% SDS, and 1% Triton X-100). Chromatin was incubated overnight at 4 °C with 5 μg of H3K27ac and FOS antibodies, and then subjected to immunoprecipitation (IP) with 50 μL of Protein A Dynabeads.

The IP beads were washed twice with low salt wash buffer (20 mM Tris-Cl (pH 8.0), 150 mM NaCl, 2 mM EDTA, 0.1% SDS, and 1% Triton X-100), once with high salt wash buffer (20 mM Tris-Cl (pH 8.0), 500 mM NaCl, 2 mM EDTA, 0.1% SDS, and 1% Triton X-100), once with LiCl wash buffer (10 mM Tris-Cl (pH 8.0), 0.25 M LiCl, 1 mM EDTA, 1% NP-40, and 1% Na-deoxycholate), and twice with 1× Tris-EDTA buffer (10 mM Tris-Cl (pH 8.0) and 1 mM EDTA).

Chromatin was eluted from the beads by adding elution buffer (100 mM sodium bicarbonate, 1% SDS) and incubating at 65°C for 1 h. Crosslinking was reversed by adding 2 μg of RNase A (Sigma-Aldrich) and 0.25 M NaCl, followed by overnight incubation at 65°C. DNA was purified using a PCR purification kit (Qiagen) following the manufacturer’s protocol.

ChIP and input DNA libraries were prepared using the NEBNext Ultra II DNA Library Prep kit (New England Biolabs; E7645), following the manufacturer’s instructions. The ChIP-seq libraries were sequenced on an Illumina NextSeq500 platform. Antibodies used for ChIP-seq are listed in Table 4.

**Table 4:** Antibodies used in this study.

Antibody (clone)	Supplier (catalog number)	Dilution/Quantity
Western blot		
β-actin (AC-15)	Santa Cruz (sc47773)	1:10,000
c-KIT (D13A2)	Santa Cruz (sc17806)	1:1000
Phospho-c-Kit (Tyr719)	Cell Signaling Technologies (3391)	1:1000
APC	Sigma-Aldrich (FE9)	1:1000
p53	Santa Cruz (sc126)	1:3000
c-FOS	Cell Signaling Technology (2250)	1:1000
c-Jun	Cell Signaling Technology (9165)	1:1000
Axin2	Proteintech (20540-1-AP)	1:1000
ERK	Cell Signaling Technology (9102 s)	1:1000
Phospho-ERK	Cell Signaling Technology (9101 s)	1:1000
MEK	Cell Signaling Technology (9122 s)	1:1000
Phospho-MEK	Cell Signaling Technology (9127 s)	1:1000
Akt	Cell Signaling Technology (9272)	1:1000
Phospho-Akt	abcam (ab38513)	1:1000
mTOR	Cell Signaling Technologies (2972)	1:1000
Phospho-mTOR	Cell Signaling Technologies (2448)	1:1000
p38	Cell Signaling Technologies (9212)	1:1000
Phospho-p38	Cell Signaling Technologies (4511)	1:1000
JNK	Cell Signaling Technologies (9252)	1:1000
Phospho-JNK	Cell Signaling Technologies (9251)	1:1000
Chromatin immunoprecipitation		
H3K27ac	Abcam (ab4729)	5 μg
Rabbit IgG	Abcam (ab37415)	5 μg
c-FOS	Cell Signaling Technology (2250)	5 μg
Immunohistochemistry		
Ki67	Invitrogen (MAS-14520)	1:300
CK20	Abcam (ab854)	1:100

### Bioinformatic analysis of ChIP-sequencing

Single-end ChIP-seq libraries for H3K27ac and FOS were generated. Reads were trimmed with Trimmomatic to remove adapter sequences and low-quality bases, then mapped to the human hg19 reference genome with Bowtie2 using the --very-sensitive option. The aligned reads were filtered to remove mitochondrial reads and PCR duplicates using SAMtools and Picard. The processed BAM files were then converted into HOMER tag directories using makeTagDirectory, serving as a unified input format for downstream analyses. Peak calling was performed using HOMER’s findPeaks tool, utilizing matched input samples as controls. ChIP-seq datasets for transcription factors (e.g., FOS) were analyzed using the -style factor parameter, while histone modification datasets (e.g., H3K27ac) were analyzed using the -style histone parameter. Peak annotation and normalized peak intensity quantification were performed using annotatePeaks.pl. All ChIP-seq experiments were performed with biological replicates.

### Enzymatic methylation sequencing (EM-seq)

Fifty ng of genomic DNA were combined with 0.1 ng CpG-methylated pUC19 and 2 ng unmethylated lambda control DNA and were made up to 50 µL with 10 mM Tris 0.1 mM EDTA (pH 8.0). DNA was sheared by sonication using a Misonix 3000 to achieve an average fragment size of 200–400 bp. Fifty microliters of sheared material were transferred to a PCR strip tube to begin library construction. NEBNext DNA Ultra II reagent (NEB) was used according to the manufacturer’s instructions. The prepared libraries were sequenced using the Illumina NovaSeq 6000 platform with 2 × 150 bp to generate approximately 100 Gb of raw sequencing data.

### Bioinformatic analysis of EM-sequencing

After paired-end reads were trimmed with Trimmomatic to remove adapter sequences and low-quality bases, they were mapped to the human hg19 reference genome using BitMapperBS. To assess enzymatic conversion efficiency, reads were aligned to unmethylated control genomes (λ-DNA and pUC19). The aligned BAM files were sorted and indexed using the same tools, and PCR duplicates were removed using Picard. Methylation calls in CpG, CHG, and CHH contexts were obtained using MethylDackel with the -CHG and -CHH options. Methylation extraction was performed for each sample against both hg19 and spike-in reference genomes; all samples exhibited low CHG and CHH methylation levels in λ-DNA and pUC19, confirming high enzymatic conversion efficiency. For differential methylation analysis, CpG sites were aggregated into 1000 bp windows using tileMethylCounts, and differentially methylated regions (DMRs) were identified using logistic regression models implemented in the methylKit package. For visualization, tracks were generated using the makeUCSCfile tool and viewed in the IGV. All Methyl-seq experiments were performed with biological replicates.

### Western blot analysis

Organoids were lysed using a lysis buffer containing 50 mM Tris-HCl (pH 7.5), 1% NP-40, 0.1% sodium deoxycholate, 150 mM NaCl, 1 mM sodium pyrophosphate, 1 mM EDTA, and protease/phosphatase inhibitors for 30 min with gentle inversion on ice. After centrifugation, the whole-cell lysates were collected. Protein concentration was measured using Bicinchoninic Acid Protein Assay reagent (Pierce, #23225) according to the manufacturer’s protocol. 10-60 µg of the sample were loaded onto an 8-12% SDS-PAGE gel. The gel was transferred to a nitrocellulose membrane and blocked for 1 h at room temperature with 5% skim milk. The membranes were then incubated with the appropriate primary antibody overnight at 4°C. Following a wash with Tris-buffered saline + Tween 20 (TBST), the membrane was incubated with HRP-conjugated secondary antibody for 1 h at room temperature. After washing with TBST, HRP-protein signals were detected using an enhanced chemiluminescence (ECL) solution. The antibodies used for western blotting are listed in Table 4.

All western blots are shown as cropped images, with raw blots provided with molecular weight/size markers labeled in Supplementary Fig. S8.

### Phospho-kinase array

To evaluate the relative phosphorylation of 43 different kinase sites, a Human Phospho-Kinase Array kit (R&D Systems, Minneapolis, MN, USA; ARY003C) was used, following the manufacturer’s instructions. Organoid lysates (200 μg) were incubated using the Phospho-Kinase Array kit. The array was treated with phospho-specific antibodies and detected by chemiluminescence, with signal enhancement using an enhanced chemiluminescence substrate.

### Xenoplantation in nude mice

For xenotransplantation, 1 × 10^6^ organoid cells resuspended in cold 50% Matrigel were injected into the right flank of female 6-week-old BALB/c nude mice (Orient Bio, Seongnam-si, Republic of Korea; *n* = 5 mice per group). For the T-5224 (Toyama Chemical, Japan) treatment assay, T5224 was diluted in PVP solution, following the development of tumors in ΔDKO mice at five months. T-5224 (150 mg/kg body weight) was administered orally to the treatment group daily for four weeks, while the control group received only the PVP solution (vehicle) (*n* = 4 mice per group). Xenograft size was measured twice a week. Tumor xenograft volume (*V*) was calculated using a caliper to measure the length (*L*) and width (*W*), applying the formula: *V* = 1/2 * (L*W^2^). The mice were sacrificed at the end of the experiment, and the xenografts were excised for IHC analysis.

There was no sample size calculation‚ but the number of animals used was based on prior experience. No animals or samples were excluded from the analysis, and no pre-established inclusion or exclusion criteria were applied. Animals were assigned to experimental groups based on genotype, and no method of randomization was used. Investigators were not blinded to group allocation during the experiments or outcome assessment.

All animal experiments were performed in accordance with the National Institutes of Health Guidelines under protocols approved by the Institutional Animal Care and Use Committee (permission number: SNU-250703-8) of the Seoul National University (Seoul, Korea).

### Statistical analyzes

No statistical methods were used to predetermine sample size․ Statistical tests used for each figure are indicated in the corresponding figure legends. Statistical significance was determined using an unpaired two-tailed Student’s *t*-test. Data are displayed as means ± standard deviation (SD). *p*-value < 0․05 was considered statistically important․ Statistical analyses were conducted using GraphPad Prism version 8 (GraphPad Software‚ Boston‚ MA‚ USA)․In all figures, the significance levels are denoted by asterisks as follows: *p < 0.05, **p < 0.01, and ***p < 0.005.

## Results

### Generation and characterization of *APC*/*TP53* double knockout colon organoids (ΔDKO) exhibiting enhanced proliferative capacity

**Fig. 1:**
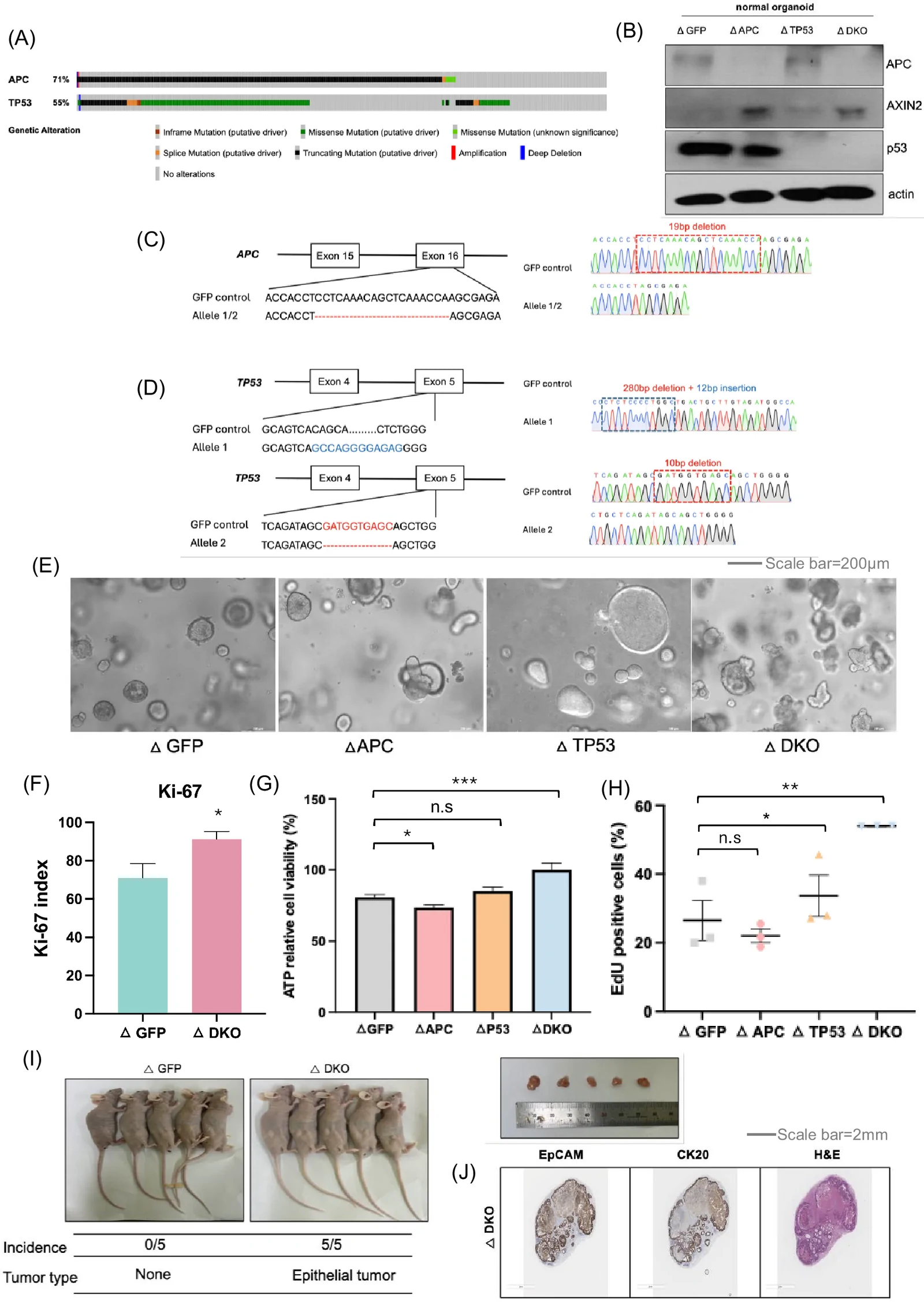
*APC*/*TP53* double knockout (ΔDKO) colorectal organoids were generated and characterized. **A** Oncoplot depicting genomic alterations in 423 colorectal cancer samples. Among 423 samples, *APC* (71%) and *TP53* (54%) mutations were frequently observed, indicating their high prevalence in tumorigenesis. **B** Western blot analysis confirming knockout of APC and TP53 in ΔGFP, Δ*APC*, Δ*TP53*, and ΔDKO organoids. Actin was used as a loading control. **C**, **D** Sanger sequencing chromatograms displaying CRISPR-induced deletions in the *APC* and *TP53* genes in a normal organoid. Red annotations indicate deletion sites and blue annotations indicate insertion sites in the DNA sequence, confirming gene knockout. **E** Representative images of ΔGFP, Δ*APC*, Δ*TP53*, and ΔDKO organoids. Scale bar = 200 µm. **F** Ki-67 index in ΔGFP and ΔDKO organoids (*n* = 3). Data are shown as mean ± SD; (*p < 0.05). **G** ATP-based cell viability assay demonstrating that ΔDKO organoids exhibit significantly enhanced viability compared to single knockouts and control organoids (*n* = 3). Data are shown as mean ± SD; (*p < 0.05, ***p < 0.001, n.s. = not significant). **H** The percentage of EdU-positive cells in ΔGFP, Δ*APC*, Δ*TP53*, and ΔDKO organoids (*n* = 3). Data are shown as mean ± SD; (*p < 0.05, **p < 0.01, n.s. = not significant). **I**, **J** organoid cells (1 × 10^6^ cells per injection) were subcutaneously injected into the right flank of nude mice (*n* = 5 mice per group). **I** Xenografts images and incidence rates after injection with organoid cells transduced with ΔGFP and ΔDKO. **J** Representative H&E, EpCAM, and CytoKeratin20 (CK20) IHC staining in xenografts derived from ΔDKO organoids.

To elucidate the mechanisms governing early colorectal tumorigenesis, we developed a colon organoid model featuring a double knockout of *APC* and *TP53*, two tumor suppressor genes that are commonly inactivated and play crucial roles in the development of colorectal cancer (CRC). Co-mutation of *APC* and *TP53* was observed in approximately 45% of CRC cases (Fig.1A), a rate higher than that of other common mutations, such as *KRAS* ( ~ 35%) (Fig. S1a) or *SMAD4* ( ~ 10%) in TCGA data (Fig. S1b). Frequently mutated driver genes in CRC, including *APC* and *TP53*, were detected in tumor tissues but not in normal tissues (Fig. S1c).

Given that *APC* loss is widely considered an initiating event in colorectal carcinogenesis [4], we first introduced sgRNAs targeting *APC* into normal human colon organoids using the CRISPR-Cas9 genome editing system. Functional selection was performed by withdrawing Wnt and R-spondin, which are key niche factors essential for the survival of the wild-type colon epithelium [22, 23]. Next, we introduced an additional *TP53* mutation to generate ΔDKO organoids and compared them with *TP53*-only knockout organoids (Δ*TP53*) as a control. We verified successful genome editing by genotyping and western blotting on clonal organoid lines derived from single organoids (Fig. 1B–D). In the *APC* region, both alleles exhibited a homozygous 19 bp deletion in exon 16, confirming complete biallelic inactivation (Fig. 1C). For *TP53*, we identified compound heterozygous mutations in exon 5, consisting of a 280 bp deletion with a 12 bp insertion in one allele and a 10 bp deletion in the other. (Fig. 1D).

To validate the constitutive activation of the Wnt signaling pathway resulting from *APC* loss, we evaluated both the growth of organoids and expression levels of the Wnt target gene *AXIN2* [24]. As expected, both GFP control (ΔGFP) and Δ*TP53* organoids showed reduced viability under Wnt/R-spondin withdrawal (EN media), whereas ΔDKO organoids retained growth similar to Δ*APC* organoids. (Fig. S1d). Consistently, *AXIN2* expression remained elevated in Δ*APC* and ΔDKO organoids even under Wnt/R-spondin-deprived conditions, indicating successful *APC* inactivation and constitutive activation of the Wnt pathway (Fig. S1e).

Next, we examined the phenotypic consequences of *APC*, *TP53*, and *APC*/*TP53* double loss in normal colon organoids. Morphologically, Δ*APC* organoids exhibited enhanced stem cell activity with a budding phenotype, whereas Δ*TP53* organoids showed an enlarged cystic morphology. Notably, ΔDKO organoids displayed a combination of both phenotypes (Fig. 1E). Histological analysis using H&E staining, along with Ki67 immunostaining, revealed elevated Ki67 intensity in ΔDKO organoids compared to ΔGFP controls (71.0 versus 91.1%) (Fig. 1F and S1f). This was further supported by ATP assay and EdU incorporation assays, indicating elevated DNA synthesis and cell proliferation in the ΔDKO organoids (ATP: 100 versus 123.9%; EdU: 26.5 versus 53.9%) (Fig. 1G, H and S1g).

To evaluate the in vivo effects of *APC*/*TP53* inactivation in normal colon organoids, we performed xenotransplantation assays. ΔGFP and ΔDKO organoid cells were subcutaneously injected into the right flank of five nude mice. Over a 5-month observation period, no tumors were observed in five mice injected with ΔGFP organoids. In contrast, ΔDKO organoids developed tumors within 20 weeks in all mice (Fig. 1I). H&E and immunohistochemistry (IHC) analysis of xenografts derived from ΔDKO organoids showed positive expression of EpCAM and CK20, confirming the epithelial tumor origin (Fig. 1J).

These findings collectively demonstrate that the concurrent loss of *APC* and *TP53* transforms normal colon organoids into highly proliferative tumorigenic structures both in vitro and in vivo.

### Coordinated epigenetic alterations and transcriptional reprogramming in ΔDKO organoids

**Fig. 2:**
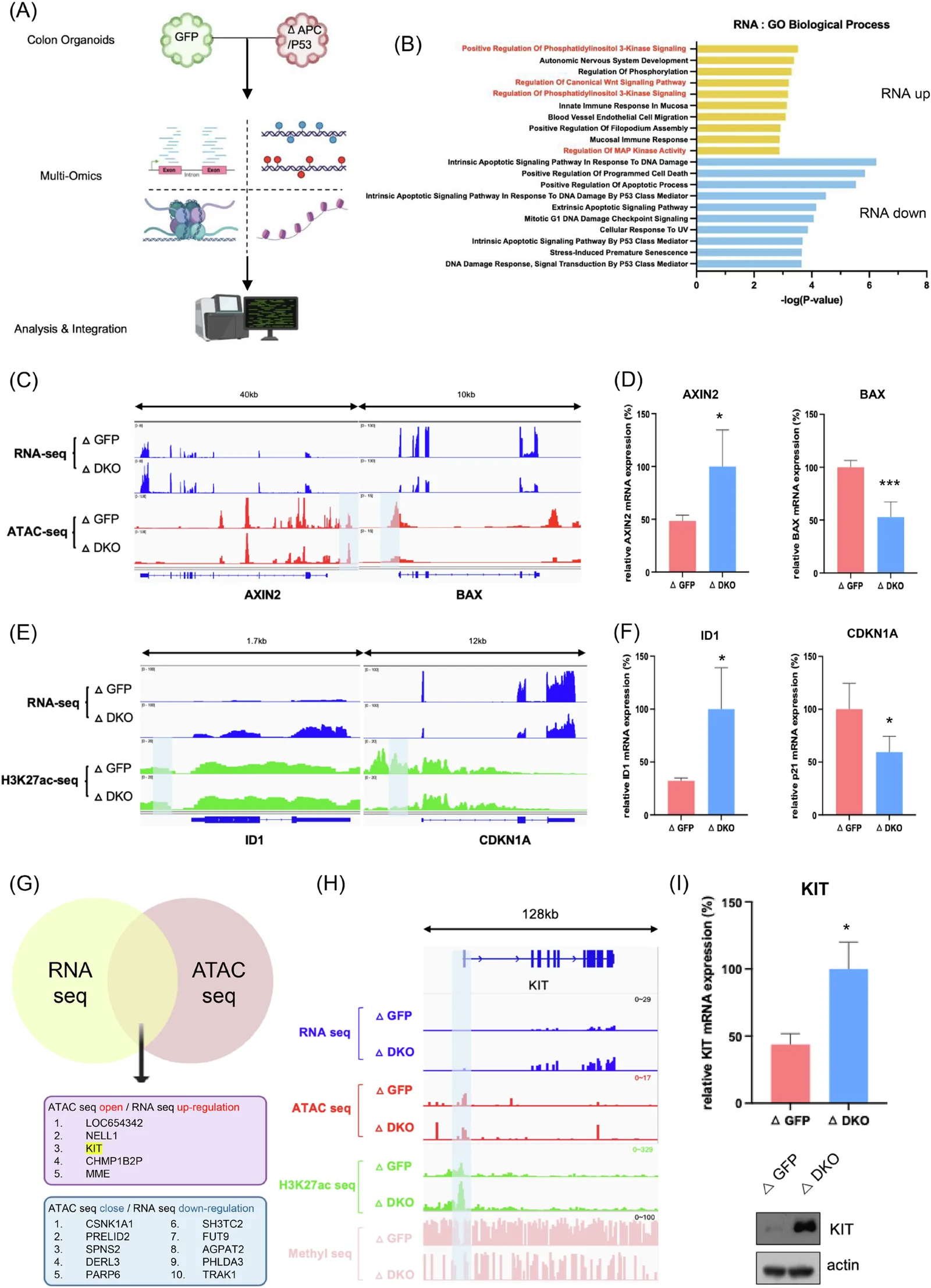
Multi-omics profiling identifies *KIT* upregulation in ΔDKO organoids. **A** Schematic overview of the multi-omics analysis performed on ΔGFP and ΔDKO colon organoids. RNA sequencing, ATAC sequencing, H3K27ac ChIP-seq, and DNA methylation sequencing were integrated to assess transcriptional, chromatin, and epigenetic changes in ΔDKO organoids compared to ΔGFP controls. **B** Top enriched Gene Ontology (GO) biological process terms for genes upregulated or downregulated in ΔDKO compared with ΔGFP. Enrichment analysis was performed using EnrichR (GO Biological Process 2025 library). Tumor-associated pathways are highlighted in red, and GO terms are ranked by enrichment. **C**–**F** RNA-seq and ATAC-seq analysis reveal key gene expression and chromatin accessibility changes in ΔDKO organoids. (c) RNA-seq and ATAC-seq tracks around *AXIN2* and *BAX* in ΔGFP and ΔDKO organoids. **D** qRT-PCR assay was performed to detect the expression of *AXIN2* and *BAX* in ΔGFP and ΔDKO organoids. **E** RNA-seq and H3K27ac-seq tracks around *ID1* and *CDKN1A* in ΔGFP and ΔDKO organoids. **F** qRT-PCR assay was performed to detect the expression of *ID1* and *CDKN1A* in ΔGFP and ΔDKO organoids. Expression normalized to 18 s, and horizontal bars represent the mean of *n* = 3 individual experiments. (*p < 0.05, ***p < 0.001). **G** Venn diagram showing the overlap of RNA-seq and ATAC-seq data, highlighting genes with coordinated transcriptional and chromatin accessibility changes (top). Genes in the pink box exhibit increased chromatin accessibility (ATAC-seq open) along with upregulation in RNA-seq, whereas genes in the blue box exhibit decreased chromatin accessibility (ATAC-seq close) along with downregulation in RNA-seq. **H** RNA-seq, ATAC-seq, H3K27ac-seq, and methyl-seq tracks around *KIT* in ΔGFP and ΔDKO organoids. **I** qRT-PCR assay was performed to detect the expression of *KIT* in ΔGFP and ΔDKO organoids (top). Western blot analysis confirming expression of KIT in ΔGFP and ΔDKO organoids. Actin was used as a loading control (bottom). Expression normalized to 18 s, and horizontal bars represent the mean of *n* = 3 individual experiments (*p < 0.05).

To investigate the dynamic changes in the genetic and epigenetic landscape during the initial stages of colon carcinogenesis, we conducted a comprehensive multi-omics profiling approach, comprising RNA-seq, ATAC-seq, H3K27ac ChIP-seq, and Methyl-seq on ΔGFP and ΔDKO colon organoids (Fig. 2A). Principal component analysis (PCA) indicated that biological replicates within the ΔGFP cohort exhibited clustering, whereas the ΔDKO cohort was markedly differentiated (data not shown). We identified 104 differentially expressed genes (DEGs), 1,096 differentially accessible regions (DARs), 407 differentially acetylated regions (DARs), and 27,883 differentially methylated regions (DMRs) between ΔGFP and ΔDKO organoids (Figs. S2a and S2b).

Gene ontology (GO) analysis identified several tumor-associated pathways, such as regulation of canonical Wnt signaling, epithelial to mesenchymal transition (EMT) and cell migration involved in sprouting angiogenesis, which were upregulated in ΔDKO organoids compared to ΔGFP controls (Fig. 2B and S2c–e). These pathways are well-known drivers of tumor progression, including CRC. For example, EMT facilitates cellular motility and invasiveness, contributing to tumor growth and metastasis [25, 26]. Likewise, canonical Wnt signaling enhances tumor progression by promoting cell proliferation and survival [27, 28]. These findings are consistent with our previous observations of enhanced proliferative capacity in ΔDKO organoids (Fig. 1F–H).

Specifically, ATAC-seq analysis showed that the majority of accessible regions were located in the intronic and intergenic regions. Notably, ΔDKO organoids exhibited an increase in accessibility at promoter regions (8% → 12.5%) (Fig. S2f). Furthermore, we observed differential patterns in the regions that gained or lost accessibility exclusively in ΔGFP or ΔDKO organoids (Fig. S2f). Similarly, H3K27ac ChIP-seq revealed distinct histone acetylation patterns in intronic and intergenic regions, with notable changes in acetylation in ΔDKO organoids compared to ΔGFP controls (Fig. S2g). These changes reflect genotype-dependent remodeling of the chromatin landscape and highlight the distinct transcriptional regulatory states in each condition.

To better understand the relationship between chromatin structure and gene expression, we analyzed genome-wide correlations. Increased RNA expression was generally associated with enhanced chromatin accessibility and H3K27ac enrichment at the corresponding loci (Fig. S2h and S2i), supporting a model in which *APC*/*TP53* co-inactivation drives global epigenetic remodeling and oncogenic transcriptional activation.

To evaluate this correlation at the gene level, we selected four genes that were expected to be upregulated or downregulated in ΔDKO organoids, based on their functional association with key pathways, including Wnt signaling (*AXIN2*) [24, 29], apoptosis (*BAX*) [30], cell differentiation (*ID1*) [31, 32], and cell cycle regulation (*CDKN1A*) [33]. As expected, *AXIN2* demonstrated increased expression and chromatin accessibility, whereas *BAX* exhibited concurrent reductions in both expression and accessibility in ΔDKO organoids (Fig. 2C). Likewise, *ID1* expression was accompanied by elevated H3K27ac levels, whereas *CDKN1A* displayed reduced expression and histone acetylation in ΔDKO organoids (Fig. 2E). These findings were further validated by qRT-PCR analysis (Fig. 2D, F), confirming the concordance between gene expression and epigenomic activation states at representative loci.

Beyond chromatin accessibility and histone modifications, we further investigated whether DNA methylation also contributes to the transcriptional landscape of ΔDKO organoids. Consistent with our transcriptomic findings, oncogenic drivers and stemness-related genes, such as *LGR5* [34], exhibited significant hypomethylation at their promoter regions, which correlated with their robust transcriptional induction. Conversely, the downregulation of the differentiation marker *CA2* [35] was associated with extensive hypomethylation within its gene body (Fig. S3a, S3b). However‚ these locus-specific changes in concordance were not recapitulated on the genome-wide level‚ and poor correlation between changes in DNA methylation and global RNA expression was observed (data not shown)‚ suggesting that DNA methylation remodeling may be a phenomenon that occurs before transcriptional reprogramming of entire networks‚ or is not always translated to changes in gene expression․

Among the genetic elements that exhibit synchronized epigenetic and transcriptional modifications, *KIT* has emerged as a significant candidate (Fig. 2G). ΔDKO organoids demonstrated significantly elevated levels of *KIT* expression, which correlated with enhanced chromatin accessibility, H3K27ac enrichment, and hypomethylation within the upstream regulatory region (Fig. 2H). This increase in expression was further substantiated by quantitative reverse transcription polymerase chain reaction (qRT-PCR) and western blot analyses (Fig. 2I). Importantly, *KIT* expression remained consistently elevated in ΔDKO organoids, regardless of exogenous Wnt/R-spondin stimulation (Figure S3c), indicating that its regulation operates independently of the canonical Wnt signaling pathway. Notwithstanding prior investigations that have associated *KIT* with colorectal cancer (CRC) tumorigenesis [36–38], its function during the initial phases of tumor development remains ambiguous, necessitating further exploration.

### Functional analysis of *KIT* in early tumorigenesis of ΔDKO organoids

**Fig. 3:**
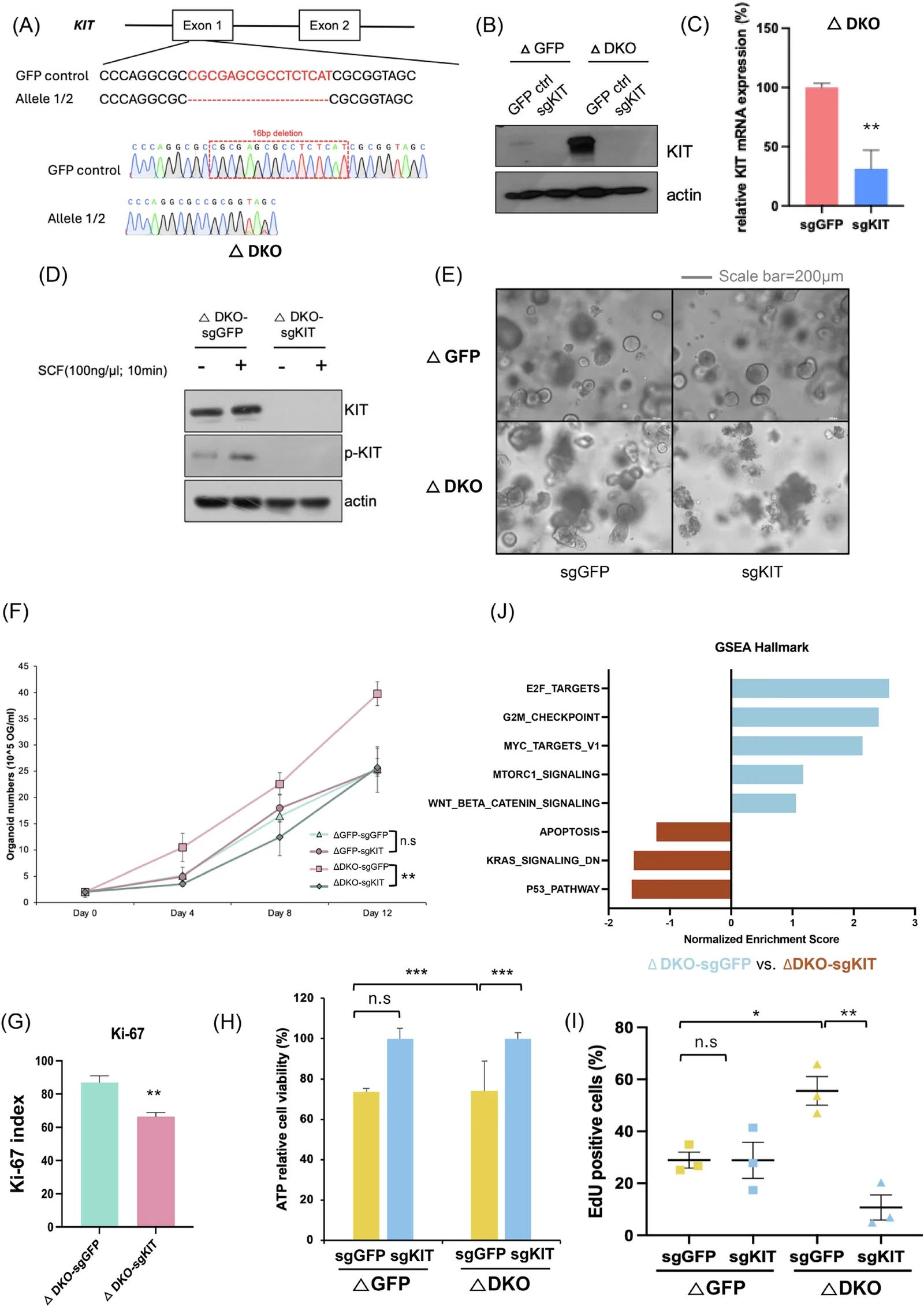
*KIT* inhibition suppresses the tumorigenic potential of ΔDKO organoids. **A** Sanger sequencing chromatogram displaying CRISPR-induced deletions in the *KIT* genes in ΔDKO organoids. Red annotations indicate 16 bp deletion sites in the DNA sequence, confirming gene knockout. **B** Western blot analysis confirming knockout of KIT (sg*KIT*) in ΔGFP and ΔDKO organoids. Actin was used as a loading control. **C** qRT-PCR assay was performed to detect the expressions of *KIT* following knockout of *KIT* in ΔDKO organoids. Expression normalized to 18 s and horizontal bars represent the mean of *n* = 3 individual experiments (**p < 0.01). **D** Western blot analysis of KIT and phosphorylated KIT upon stimulation with stem-cell factor (SCF, 100 ng/µL) for 10 min in ΔDKO-sgGFP and ΔDKO-sg*KIT*. **E** Representative images of ΔGFP and ΔDKO organoids with *KIT* knockout. Scale bar = 200 µm. **F** Organoid growth curves for ΔGFP and ΔDKO with sgGFP or sg*KIT* (*n* = 3). Statistical significance was determined by one-way ANOVA (**p < 0.01, n.s. = not significant). **G** Ki-67 index in ΔGFP and ΔDKO organoids (*n* = 3). Data are shown as mean ± SD; (**p < 0.01). **H** ATP-based cell viability assay for ΔGFP and ΔDKO organoids with sgGFP or sg*KIT* (*n* = 3). Data are shown as mean ± SD; (***p < 0.001, n.s. = not significant). **I** The percentage of EdU-positive cells in ΔGFP and ΔDKO organoids with sgGFP or sg*KIT* (*n* = 3). Data are shown as mean ± SD; (*p < 0.05, **p < 0.01, n.s. = not significant). **J** Gene set functional enrichment analysis was performed using gProfiler2. Gene set enrichment analysis for ΔDKO organoids with sgGFP or sg*KIT* (*n* = 2), resulting in differential Hallmark signaling pathways.

To investigate the role of *KIT* in early colorectal tumorigenesis, we applied CRISPR-Cas9-mediated knockout of *KIT* in ΔDKO organoids by targeting exon 1. Genotyping of clonal single organoids revealed a 16 bp homozygous deletion, and both western blotting and qRT-PCR confirmed the loss of *KIT* expression in ΔDKO organoids (Fig. 3A–C). Moreover, SCF stimulation induced phosphorylation of KIT at Tyr719 in ΔDKO organoids, whereas no phosphorylation was detected in *KIT*-depleted organoids (Fig. 3D), confirming effective functional inactivation.

To assess the effect of *KIT* loss in each genetic background, we knocked out *KIT* in both ΔGFP and ΔDKO organoids and evaluated the resulting phenotypes in each context. Morphologically, *KIT* depletion had no observable effect on ΔGFP organoids, which retained their cystic structure and number of organoids (Fig. 3E, F). In contrast, *KIT*-depleted ΔDKO organoids exhibited a 23% reduction in the number and increased aggregation (Fig. 3E, F). Consistent with the observed morphological changes, Ki67 immunostaining revealed a 20% reduction in *KIT*-depleted ΔDKO organoids (Fig. 3G and S4a). This was accompanied by a 28% decrease in overall viability (Fig. 3H) and a pronounced reduction in DNA synthesis, as shown by EdU incorporation (55 versus 10%) (Fig. 3I and S4b). In contrast, *KIT* depletion had a minimal impact on the viability or proliferation of ΔGFP organoids (Fig. 3H, I).

To elucidate the molecular mechanisms underlying the observed phenotypic effects, we performed RNA sequencing of ΔDKO organoids transduced with either the control (sgGFP) or *KIT*-targeting sgRNA (sg*KIT*). Differential expression analysis identified 826 upregulated genes and 1,064 downregulated genes in *KIT*-depleted organoids relative to the control group (Fig. S4c). Gene set enrichment analysis (GSEA) revealed that *KIT*-expressing ΔDKO organoids exhibited upregulation of multiple oncogenic pathways, including E2F_TARGETS, G2M_CHECKPOINT, MYC_TARGETS_V1, MTORC1_SIGNALING, and WNT_BETA_CATENIN_SIGNALING, whereas *KIT-*depleted ΔDKO organoids were enriched for tumor-suppressive signatures, such as APOPTOSIS, KRAS_SIGNALING_DN, and P53_PATHWAY (Fig. 3J and Fig. S4d–g).

Collectively, these findings suggest that *KIT* plays an essential role in sustaining the enhanced proliferative phenotype of *APC/TP53*-deficient colon organoids by activating oncogenic transcriptional programs.

### *KIT*-dependent regulation of MAPK phosphorylation and Wnt signaling in ΔDKO organoids

**Fig. 4:**
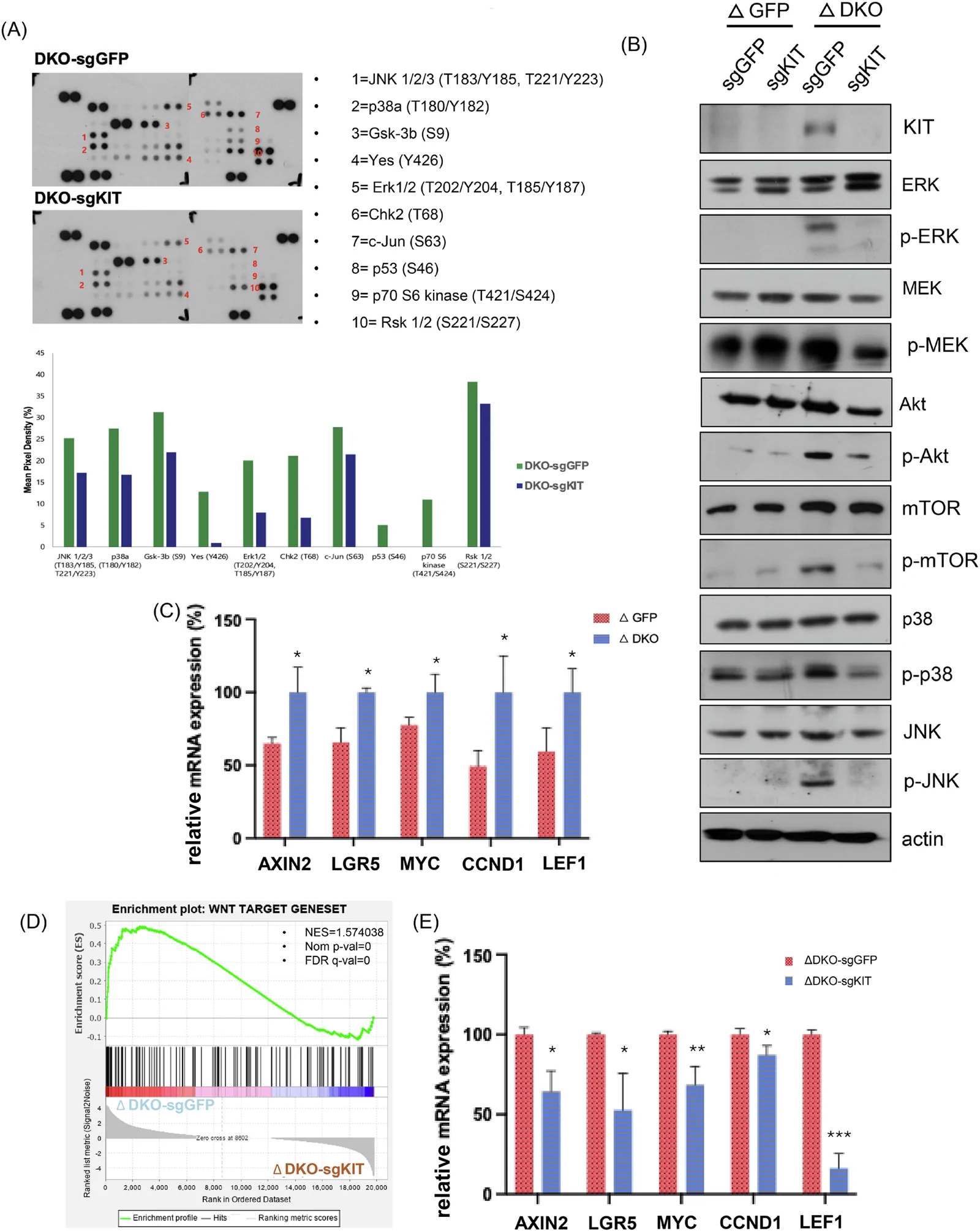
*KIT* depletion suppresses MAPK and Wnt pathway activity in ΔDKO organoids. **A** Human Phospho-Kinase Array analyzing phosphorylation levels of 43 kinases in ΔDKO with either control (sgGFP) or *KIT* knockout (sg*KIT*). The arrays display phosphorylation status of key kinases, including JNK, p38α, GSK-3β,Yes, ERK1/2, Chk2, c-JUN, p53, p70 S6 kinase, and RSK1/2 (top). Quantification of mean pixel density in the bar graph highlights significant reductions in those kinases' phosphorylation levels in ΔDKO-sg*KIT* organoids compared to ΔDKO-sgGFP organoids (bottom). **B** Western blotting showing the KIT, ERK, p-ERK, MEK, p-MEK, AKT, p-Akt, mTOR, p-mTOR, p38, p-p38, JNK and p-JNK protein levels from ΔGFP and ΔDKO organoids with or without KIT. **C** qRT-PCR assay showing upregulation of *AXIN2, LGR5, MYC, CCND1*, and *LEF1* mRNA levels in ΔGFP and ΔDKO organoids. Expression normalized to 18 s, and horizontal bars represent the mean of *n* = 3 individual experiments (*p < 0.05). **D** Enrichment plots showing expression of ΔDKO-sgGFP significantly and positively correlated with the Wnt signaling pathway. **E** qRT-PCR assay showing downregulation of *AXIN2, LGR5, MYC, CCND1*, and *LEF1* mRNA levels upon *KIT* depletion in ΔDKO organoids. Expression normalized to 18 s and horizontal bars represent the mean of *n* = 3 individual experiments (*p < 0.05, **p < 0.01, *** *p* < 0.001).

*KIT* is a receptor tyrosine kinase known to activate intracellular signaling pathways involved in cell proliferation, adhesion, apoptosis, survival, and differentiation, primarily through MAPK and PI3K/AKT cascades [36, 39–42]. To investigate its downstream effects in the context of *APC/TP53* co-inactivation, we performed a human phospho-kinase array analysis on *KIT-*depleted ΔDKO organoids. Loss of *KIT* led to a reduction in the phosphorylation of several key signaling proteins, including JNK 1/2/3 (T183/Y185, T221/Y223), p38α (T180/Y182), GSK-3β (S9), Yes (Y426), Erk1/2 (T202/Y204, T185/Y187), Chk2 (T68), c-JUN (S63), p53 (S46), p70 S6 kinase (T421/S424), and Rsk 1/2 (S221/S227) (Fig. 4A). These findings were validated by western blotting (Fig. 4B), which confirmed the broad suppression of MAPK-associated signaling pathways upon *KIT* loss.

In addition to the MAPK signaling cascade, we further investigated whether *KIT* influences the dynamics of the Wnt pathway, given its well-established role in sustaining intestinal stem cell functionality and the advancement of colorectal cancer [43, 44]. The expression levels of Wnt target genes, such as *AXIN2, LGR5, MYC, CCND1*, and *LEF1* [24, 45–48], were markedly elevated in ΔDKO organoids relative to ΔGFP control organoids (Fig. 4C). Consistently, the knockout of *KIT* in ΔDKO organoids led to a reduction in the expression of these Wnt target genes (Fig. 4D, E), implying that *KIT* plays a role in augmenting Wnt pathway activity, as observed within the *APC/TP53*-deficient framework.

Together, these results indicate that *KIT* acts as a positive regulator of both the MAPK phosphorylation and Wnt pathways, thereby supporting the hyperproliferative and stem-like phenotype of ΔDKO organoids.

### Effects of *KIT* overexpression on oncogenic signaling and proliferation in normal colon organoids

**Fig. 5:**
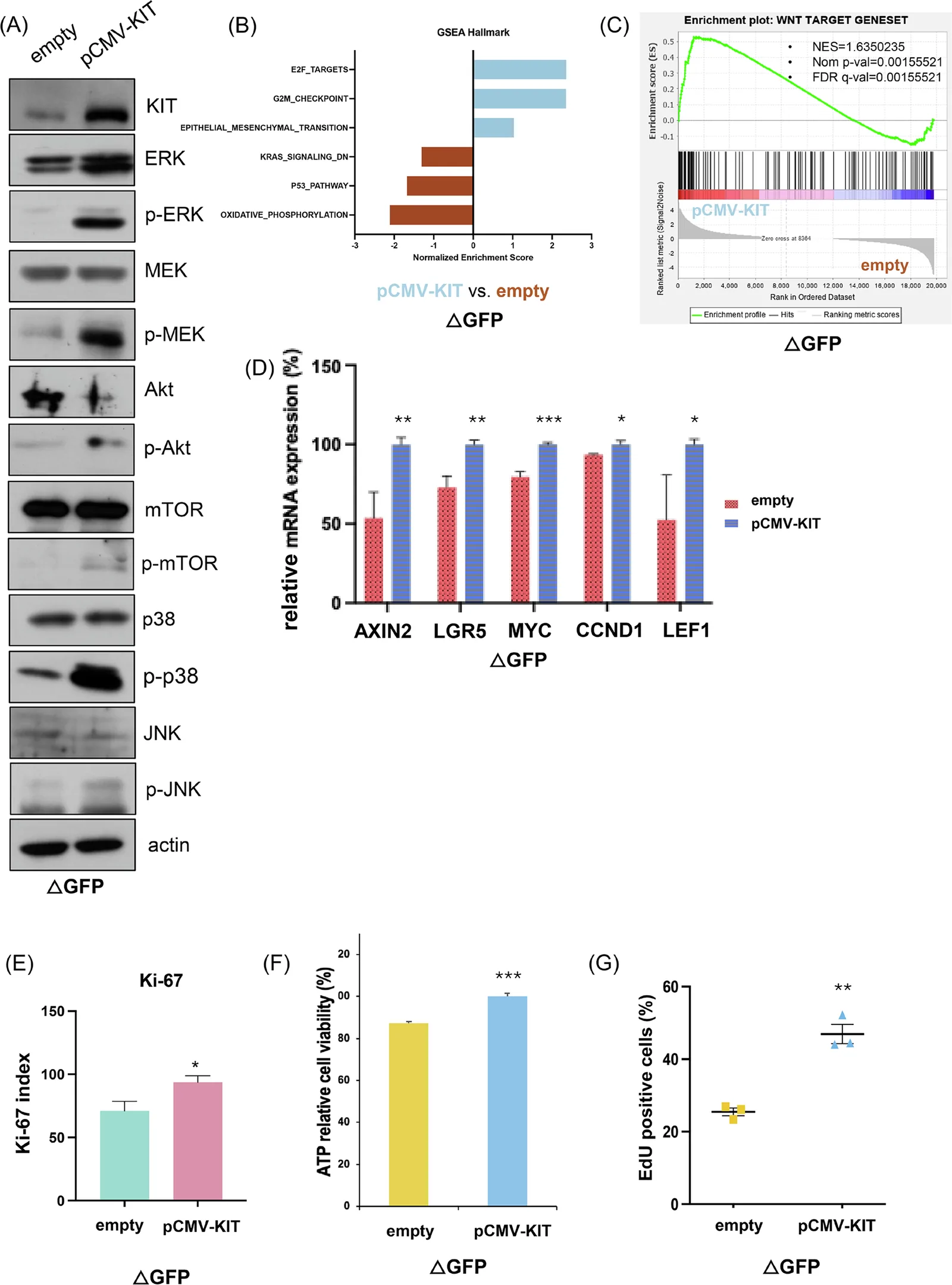
*KIT* overexpression promotes tumorigenic potential in normal colon organoids. **A** Western blotting showing the KIT, ERK, p-ERK, MEK, p-MEK, AKT, p-Akt, mTOR, p-mTOR, p38, p-p38, JNK, and p-JNK protein levels from ΔGFP organoids with or without KIT. For each phosphorylated protein, the corresponding total protein was analyzed using the same membrane. **B** Gene set functional enrichment analysis was performed using gProfiler2. Gene Set Enrichment Analysis for ΔGFP organoids with empty or pCMV-*KIT* vector (*n* = 2), resulting in differential Hallmark signaling pathways. **C** Enrichment plots showing expression of pCMV-*KIT* significantly and positively correlated with the Wnt signaling pathway. **D** qRT-PCR assay showing upregulation of *AXIN2, LGR5, MYC, CCND1*, and *LEF1* mRNA levels upon *KIT* overexpression in ΔGFP organoids. Expression normalized to 18 s and horizontal bars represent the mean of *n* = 3 individual experiments (*p < 0.05, **p < 0.01, ***p < 0.001). **E** Ki-67 index after overexpression of *KIT* in ΔGFP organoids (*n* = 3). Data are shown as mean ± SD; (*p < 0.05). **F** ATP-based cell viability assay for ΔGFP organoids with *KIT* overexpression (*n* = 3). Data are shown as mean ± SD; (***p < 0.001). **G** The percentage of EdU-positive cells in ΔGFP organoids with *KIT* overexpression (*n* = 3). Data are shown as mean ± SD; (**p < 0.01).

Next, we evaluated whether overexpression of *KIT* augments the tumorigenic capabilities of normal colon organoids. To accomplish this, we ectopically transfected the pCMV-*KIT* expression vector into ΔGFP control organoids. Overexpression of KIT resulted in modest activation of oncogenic signaling pathways, including those related to MAPK and its downstream effectors (Fig. 5A).

To assess the significant transcriptional outcomes of *KIT* upregulation, we carried out RNA sequencing in conjunction with GSEA. A total of 702 genes were upregulated, and 506 were downregulated in *KIT*-overexpressing organoids compared to empty vector controls (Fig. S5a). Enriched gene sets included hallmark oncogenic pathways such as E2F_TARGETS, G2M_CHECKPOINT, and EPITHELIAL_MESENCHYMAL_TRANSITION in *KIT*-overexpressing organoids compared to empty vector controls (Fig. 5B and S5b–e). Notably, the expression of WNT target genes, including *AXIN2, LGR5, MYC, CCND1*, and *LEF1* [24, 45–48] – was also elevated in *KIT-*overexpressing ΔGFP organoids, indicating enhanced WNT pathway activity (Fig. 5C, D). Functionally, KIT overexpression enhanced cellular proliferation, as evidenced by an increase in Ki67 index (71 versus 93.4%), viability (100 versus 114.6%), and EdU incorporation (25.5 versus 46.9%) in ΔGFP organoids (Fig. 5E–G and Fig. S5f, g).

Collectively, these findings demonstrate that ectopic *KIT* expression can activate tumor-associated transcriptional programs and promote proliferation, even in normal colon organoids and *KIT*-low colorectal cancer cells.

### AP-1-dependent *KIT* regulation via chromatin remodeling in early colon tumorigenesis

**Fig. 6:**
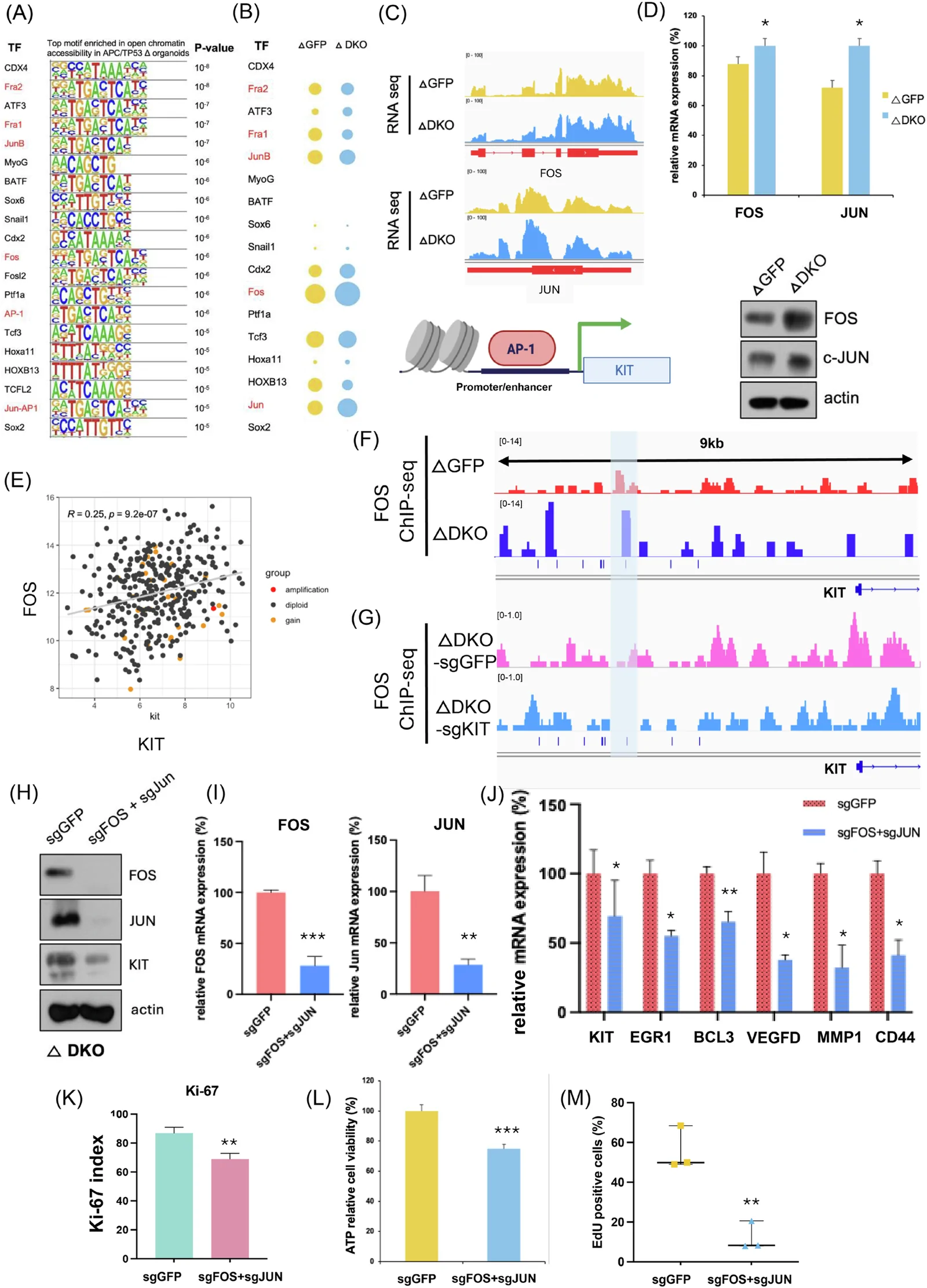
AP-1/*KIT* axis maintains proliferative capacity through chromatin-mediated transcriptional activation in early colorectal tumorigenesis. **A** Top-ranked transcription factor (TF) binding motif enriched in open chromatin accessibility in ΔDKO versus ΔGFP. *p*-values were calculated using the HOMER package. **B** Dot plots showing expression of top-ranked TFs in ΔDKO versus ΔGFP based on RNA-seq FPKM values. The dot size denotes FPKM values; yellow color indicates ΔGFP, and blue color indicates ΔDKO; missing dots correspond to undetectable mRNA expression. **C** RNA-seq tracks around *FOS* and *JUN* in ΔGFP and ΔDKO organoids (Top). Schematic illustration of a proposed mechanism by which AP-1 regulates *KIT* expression (bottom). **D** qRT-PCR assay was performed to detect the expressions of *FOS* and *JUN* in ΔGFP and ΔDKO organoids (top). Western blot analysis confirming expression of *FOS* and JUN in ΔGFP and ΔDKO organoids (bottom). Expression normalized to 18 s, and horizontal bars represent the mean of *n* = 3 individual experiments (*p < 0.05). **E** Scatterplot showing correlation between *FOS* and *KIT* expression levels. **F** ChIP-seq signal tracks for FOS around *KIT* locus in ΔGFP and ΔDKO organoids. All ChIP-seq signals are shown at the same scale. **G** ChIP-seq signal tracks for FOS around *KIT* locus ΔDKO with sgGFP or sg*KIT* organoids. All ChIP-seq signals are shown at the same scale. **H** Western blot analysis confirming silencing of FOS, JUN and KIT following knockout of *FOS* and *JUN* in ΔDKO organoids. Actin was used as a loading control. **I** qRT-PCR assay was performed to detect the expression of *FOS* and *JUN* following knockout of *FOS* and *JUN* in ΔDKO organoids. Expression normalized to 18 s and horizontal bars represent the mean of *n* = 3 individual experiments (**p < 0.01, ***p < 0.001). **J** qRT-PCR assay showing downregulation of *KIT*, *EGR1, BCL3, VEGFD, MMP1*, and *CD44* mRNA levels upon *FOS* and *JUN* depletion in ΔDKO organoids. Expression normalized to 18 s and horizontal bars represent the mean of *n* = 3 individual experiments (*p < 0.05, **p < 0.01). **K** Ki-67 index after knockout of *FOS* and *JUN* in ΔDKO organoids (*n* = 3). Data are shown as mean ± SD; (**p < 0.01). **L** ATP-based cell viability assay for ΔDKO organoids with sgGFP or sg*FOS*+sg*JUN* (*n* = 3). Data are shown as mean ± SD; (***p < 0.001). **M** The percentage of EdU-positive cells in ΔDKO organoids with sgGFP or sg*FOS*+sg*JUN* (*n* = 3). Data are shown as mean ± SD; (**p < 0.01).

DNA open chromatin regions are known to harbor regulatory elements, including transcription factor (TF)-binding sites that influence gene expression [49–51]. To identify candidate TFs driving epigenetic reprogramming in ΔDKO organoids, we performed motif analysis of differentially accessible regions from ATAC-seq data using the HOMER package [52]. Accessible peaks were enriched for consensus-binding motifs of the AP-1 transcription factor family members, including *FOSL1, FOSL2, FOS, JUN*, and *JUNB* in ΔDKO organoids (Fig. 6a).

To investigate whether AP-1 activity was upregulated, we examined the expression levels of AP-1 components and discovered that *FOS* and *JUN* were significantly increased in ΔDKO organoids, as indicated by RNA-seq, qRT-PCR, and western blot analysis (Fig. 6B–D). Analysis of TCGA data revealed a positive correlation between *FOS* and *KIT* expression in colorectal cancer (CRC) (Fig. 6E), implying that AP-1 may exert regulatory control over *KIT* transcription. In addition, *KIT*, *FOS* and *JUN* mRNA expression was evaluated in ΔGFP, ΔAPC and ΔDKO organoids (Fig. S6a). These experiments showed that a single knockout of *APC* increased expression of *KIT*, *FOS* and *JUN* mRNA in colon organoids, and this effect was further enhanced by double knockout of *APC*/*TP53*.

To assess whether AP-1 binds directly to the *KIT* locus, we performed ChIP-seq using an anti-FOS antibody in ΔGFP and ΔDKO organoids. We identified 95,810 FOS-binding peaks across the genome (Fig. S6b), with notable enrichment of FOS binding at a specific upstream regulatory region of *KIT* in the ΔDKO organoids (Fig. 6F). Consistent with this, H3k27ac ChIP-seq data also revealed strong H3k27ac signals in the same region, indicating the direct regulation of *KIT* transcription by FOS (Fig. 2H). GO analysis of ΔDKO-only FOS-bound regions revealed enrichment in pathways, such as sprouting angiogenesis and adherent junction organization, which are associated with tumor progression (Fig. S6c) [53]. Similarly, FOS binding to the same upstream regulatory region of *KIT* was reduced in *KIT*-depleted organoids, supporting the role of AP-1 occupancy in regulating *KIT* expression (Fig. 6G).

Next, to validate the role of AP-1 in regulating *KIT* expression, we deleted *JUN* and *FOS* in ΔDKO organoids (Fig. 6H, I). Loss of AP-1 activity resulted in a substantial reduction in *KIT* expression as well as decreased expression of known AP-1 target genes, such as *EGR1, BCL3, VEGFD, MMP1*, and *CD44* (Fig. 6J) [54]. Functionally, AP-1 knockout in ΔDKO organoids led to a marked reduction in cell proliferation as measured by Ki67 index (87 versus 66.5%), cell survival (100 versus 74.8%), and EdU incorporation (55.8 versus 12.3%) (Fig. 6K–M and Fig. S6d, S6e), mimicking the effects observed upon *KIT* blockade in ΔDKO organoids (Fig. 3G–I).

Taken together, these data demonstrated that AP-1 directly binds to the *KIT* regulatory region and promotes its transcription through chromatin remodeling, thereby sustaining the proliferative phenotype of ΔDKO organoids. This highlights the AP-1/*KIT* axis as a key regulatory module in early colorectal tumorigenesis and as a potential target for therapeutic intervention.

### Pharmacological inhibition of AP-1 suppresses the neoplastic properties of ΔDKO organoids

We previously demonstrated that elevated *FOS* and *JUN* expression and their direct binding to the *KIT* regulatory region contribute to the tumorigenic phenotype of ΔDKO organoids (Fig. 6). To further evaluate the therapeutic potential of pharmacological AP-1 inhibition, we treated ΔGFP and ΔDKO organoids with T-5224 1 μM, a selective AP-1 inhibitor [55, 56].

**Fig. 7:**
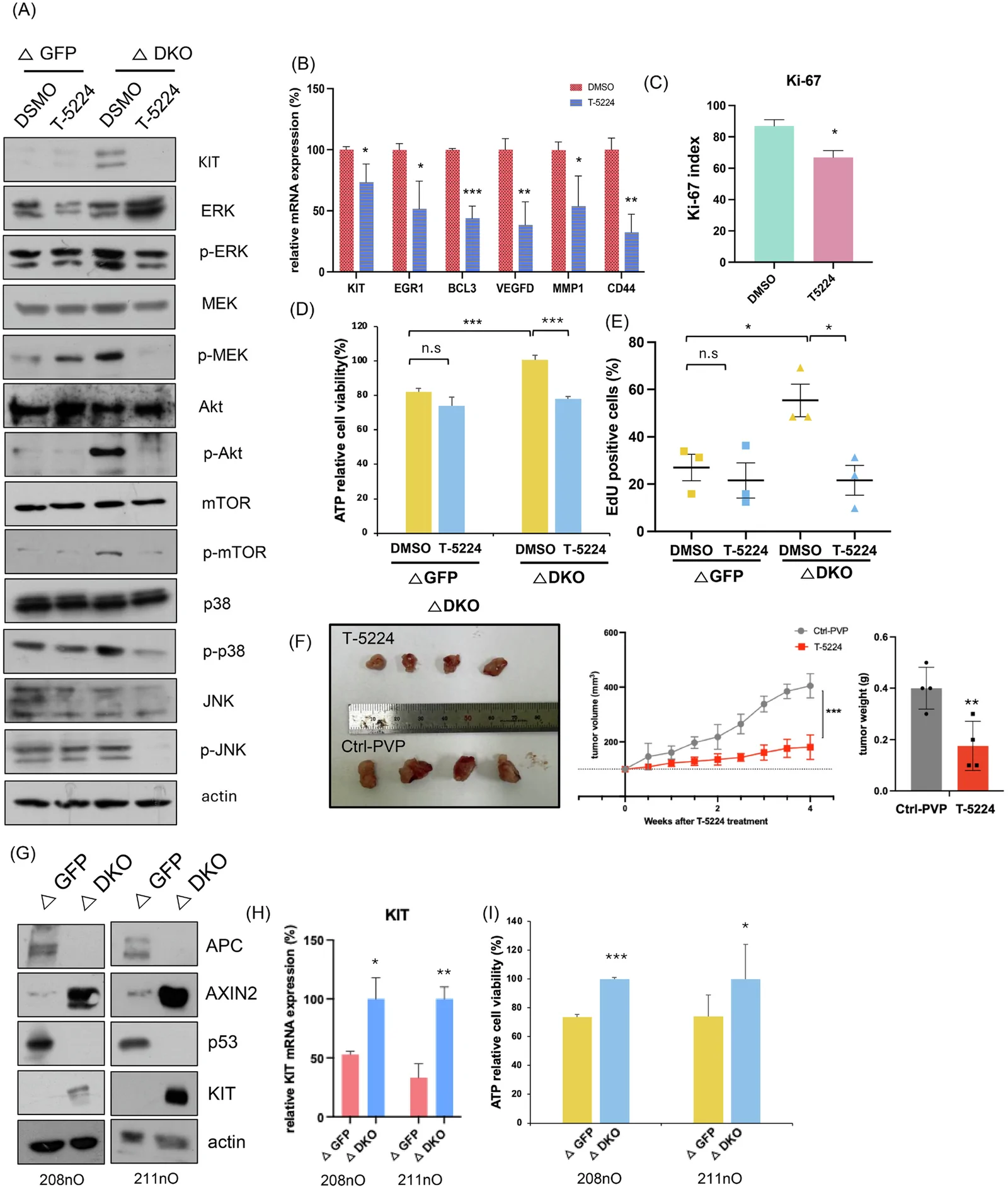
T-5224 treatment suppresses AP-1 activity and downstream tumorigenic features in ΔDKO Organoids. **A** Western blotting showing KIT, ERK, p-ERK, MEK, p-MEK, AKT, p-Akt, mTOR, p-mTOR, p38, p-p38, JNK and p-JNK protein levels from ΔGFP and ΔDKO organoids with 1μM T-5224 treatment. **B** qRT-PCR assay showing downregulation of *KIT, EGR1, BCL3, VEGFD, MMP1*, and *CD44* mRNA levels upon 1μM T-5224 treatment in ΔDKO organoids. Expression normalized to 18 s and horizontal bars represent the mean of *n* = 3 individual experiments (*p < 0.05, **p < 0.01, ***p < 0.001). **C** Ki-67 index after 1μM T-5224 treatment in ΔDKO organoids (*n* = 3). Data are shown as mean ± SD; (*p < 0.05). **D** ATP-based cell viability assay for ΔGFP and ΔDKO organoids with 1μM T-5224 treatment (*n* = 3). Data are shown as mean ± SD; (***p < 0.001, n.s.=not significant). **E** The percentage of EdU-positive cells in ΔGFP and ΔDKO organoids with 1μM T-5224 treatment (*n* = 3). Data are shown as mean ± SD; (*p < 0.05, n.s. = not significant). **F** Xenografts images after T-5224 treatment in ΔDKO organoids (*n* = 4 mice per group; left panel). Tumor volume and weight at week 4 and growth of ΔDKO xenografts with and without T-5224 treatment (right panel). Data are represented as means ± SD; **p < 0.01, ***p < 0.001 versus Ctrl-PVP on the same day by Student’s *t*-test. **G** Western blot analysis confirming knockout of APC and TP53 in ΔGFP and ΔDKO organoids derived from patients 208 and 211. **H** qRT-PCR assay was performed to detect the expression of *KIT* in ΔGFP and ΔDKO organoids derived from patients 208 and 211. Expression normalized to 18 s and horizontal bars represent the mean of *n* = 3 individual experiments (*p < 0.05, **p < 0.01). **I** ATP-based cell viability assay for ΔGFP and ΔDKO organoids derived from patients 208 and 211 (*n* = 3). Data are shown as mean ± SD; (*p < 0.05, ***p < 0.001).

Treatment with T-5224 markedly reduced the protein expression of KIT in ΔDKO organoids, but not in ΔGFP controls (Fig. 7A), which was accompanied by decreased phosphorylation of ERK, MEK, p38, Akt, and mTOR, indicating selective suppression of key oncogenic signaling pathways in the ΔDKO organoids (Fig. 7A). Consistent with the effects observed upon *FOS* and *JUN* knockout (Fig. 6H–M), qRT-PCR analysis confirmed the decreased mRNA expression of *KIT* and other AP-1 target genes (Fig. 7B) [54]. Functionally, ΔDKO organoids displayed a decrease in Ki67 index (82.5 versus 61.7%), viability (100 versus 77.9%), and EdU incorporation (55.4 versus 21.6%) after T-5224 treatment (Fig. 7C–E and Fig. S7a, b). We next assessed the effect of T-5224 in a xenograft model derived from ΔDKO. AP-1 inhibition resulted in suppression of ΔDKO xenograft growth and proliferation, indicating that T-5224 exerts significant anti-tumor effects in vivo as well (Fig. 7F).

To validate the reproducibility of these findings, we assessed independent patient-derived organoid samples (208 and 211 ΔDKO) and observed similarly elevated *KIT* expression and enhanced growth, although the magnitude of these effects varied between samples (Fig. 7G–I). Consistent with these results, RNA-seq analysis of the 208 ΔDKO organoids revealed enrichment of pathways, such as G2M_CHECKPOINT and TGF_BETA_SIGNALING (Fig. S7c), further supporting the observed phenotypic changes.

Together, these results highlight the central role of AP-1 in sustaining oncogenic signaling and *KIT* expression in *APC*/*TP53*-deficient colorectal organoids. This implies that pharmacological inhibition of AP-1 may present a feasible approach for targeting nascent tumorigenic pathways in colorectal cancer.

## Discussion

Receptor tyrosine kinases (RTKs) are frequently implicated in cancer progression, with members such as *EGFR* and *HER2* playing pivotal roles in tumorigenesis in multiple cancer types [57, 58]. *KIT*, another RTK, has been linked to stromal remodeling and tumor development [59–61]. For example, *KIT* has been shown to regulate diverse cellular processes, including proliferation, survival, migration, differentiation, and secretion in different biological contexts [60–62]. Although amplification and activating point mutations in *KIT* are well documented in gastrointestinal stromal tumors (GISTs) and melanoma [60, 62], the role of *KIT* during the initial stages of neoplastic transformation in CRC remains poorly characterized.

AP-1, a well-known regulator of tumor biology in several solid cancers, including TNBC, classical Hodgkin’s lymphoma, and ALCL [63–65], has not been extensively studied in the context of early CRC. In this study, we provide the first evidence that *KIT* activation is a critical early event in CRC driven by *APC*/*TP53* co-inactivation. Mechanistically, *APC*/*TP53* loss induces chromatin remodeling and histone acetylation at the *KIT* locus through the upregulation of AP-1 transcription factors, which directly bind to its regulatory region and promote *KIT* transcription. This, in turn, activates the downstream MAPK and Wnt signaling pathways and establishes a broader oncogenic program during early colorectal tumorigenesis.

From a therapeutic perspective, we used T-5224, a selective AP-1 inhibitor previously tested in clinical trials for arthritis [66], to assess the functional relevance of the AP-1/KIT axis. T-5224 inhibited AP-1–DNA binding [67], and its administration led to a reduction in *KIT* expression and mitigated neoplastic characteristics in ΔDKO organoids (Fig. 7A–F). Similar phenotypic suppression was observed with the genetic inhibition of *AP-1* or *KIT*, supporting the therapeutic potential of targeting the AP-1/KIT axis (Fig. 3 and 6H–M).

Interestingly‚ analysis of the TCGA dataset‚ as well as our observations on paired normal and tumor organoids derived from the same patients‚ showed that KIT is expressed at lower levels in the CRC tumor than the normal tissue (Fig. S7d–f). These findings support the possibility that KIT upregulation is more relevant to early tumor initiation than to the maintenance of established CRC. One possible explanation is that KIT expression is not retained during tumor progression, potentially in association with broader changes in tumor cell state, including dedifferentiation [68]. In line with this interpretation, a small number of differentially expressed genes identified after *APC/TP53* co-inactivation (Fig. S2a, S2b) may reflect the early-stage nature of this model. Although transcript-level changes were limited, ΔDKO organoids exhibited broader epigenomic alterations, including widespread DNA methylation changes. Because these methylation changes were not uniformly accompanied by corresponding gene-expression changes, our data suggest that DNA methylation remodeling may precede or only partially translate into more extensive transcriptional reprogramming during early colorectal tumor progression.

Our investigation is not without its limitations. Although organoid models recapitulate the key features of the native epithelium, they lack stromal and immune components present in vivo [69]. Additionally, the relatively small number of organoids limited the generalizability of our findings. Future investigations should assess whether inhibition of the AP-1/KIT axis reliably demonstrates efficacy across a diverse array of patient-derived colorectal cancer models, and whether its therapeutic potential might be enhanced in combination with currently available targeted or immunotherapeutic approaches. Moreover, given the robust upregulation of AP-1 and *KIT* in pre-malignant settings, future studies should explore their potential utility as early detection biomarkers, including through liquid biopsy-based assessment of circulating mRNA or protein levels, and further define the temporal dynamics of KIT expression during tumor progression [70, 71].

In conclusion, our work revealed a novel epigenetic mechanism through which the co-inactivation of *APC* and *TP53* potentiates *KIT* via AP-1-mediated chromatin remodeling. These findings underscore the premise that genetic mutations can fundamentally restructure the epigenomic framework to facilitate transcriptional programs that promote tumorigenesis. The AP-1/KIT axis thus serves as a mechanistic link between genetic and epigenetic dysregulation in the early stages of colorectal cancer and emphasizes a promising pathway for both early detection and therapeutic strategies.

## Supplementary information

original data

supplementary figures

## Data Availability

All sequencing data in this study were deposited in the European Nucleotide Archive (ENA) database under the accession number PRJEB89866.
