# Supplementary material for: Activation of KIT signaling promotes early tumorigenesis through the AP-1 pathway in APC/TP53 double-knockout human colon organoids
Source: Cell Death Dis. 2026 Jul 4;17(1):829. doi: 10.1038/s41419-026-09056-7 (PMC13612835; doi:10.1038/s41419-026-09056-7)

Figure 1

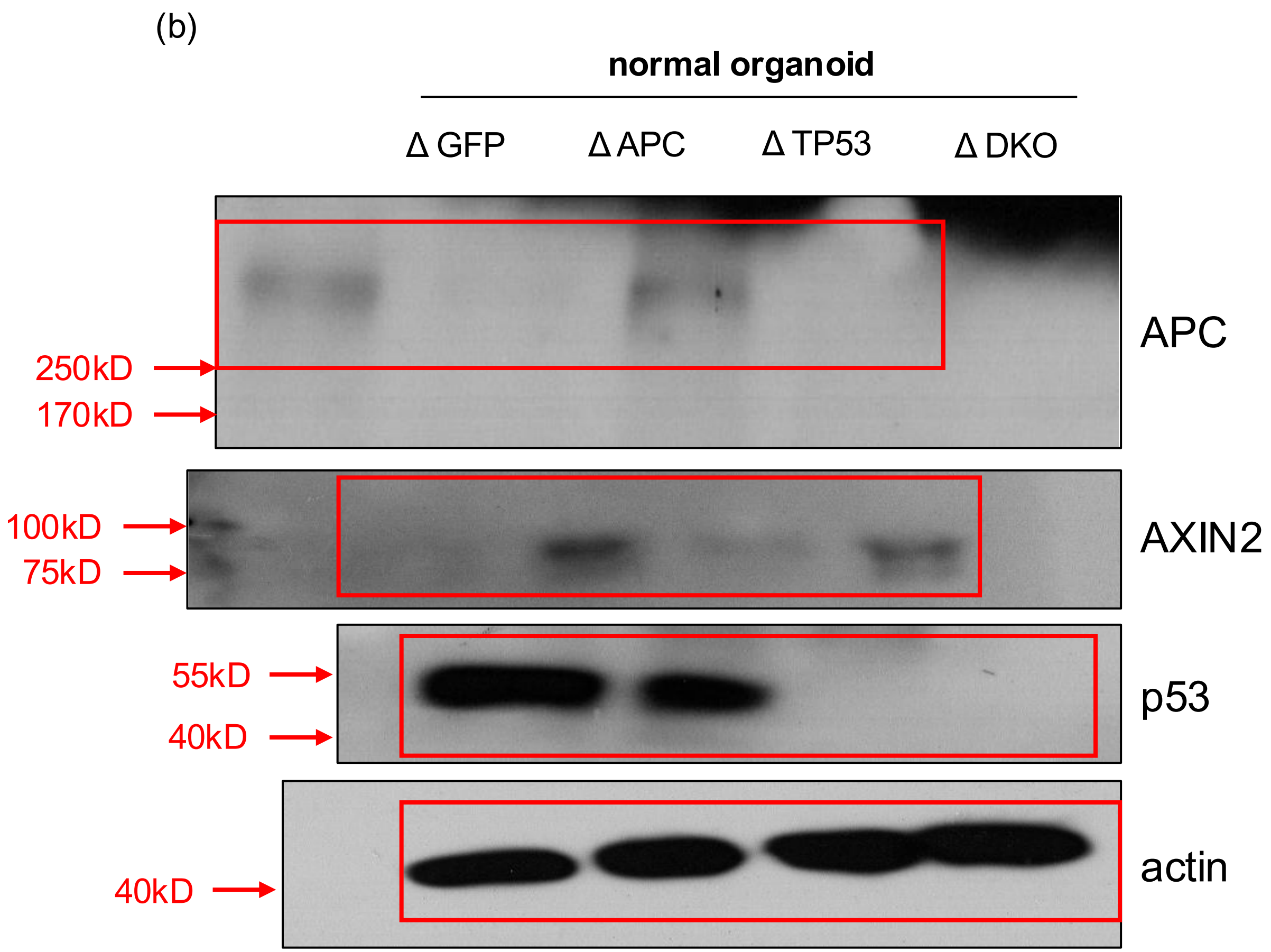
(b)
normal organoid
Δ GFP
Δ APC
Δ TP53
Δ DKO
250kD
170kD
APC
100kD
75kD
AXIN2
55kD
40kD
p53
40kD
actin

Figure 2

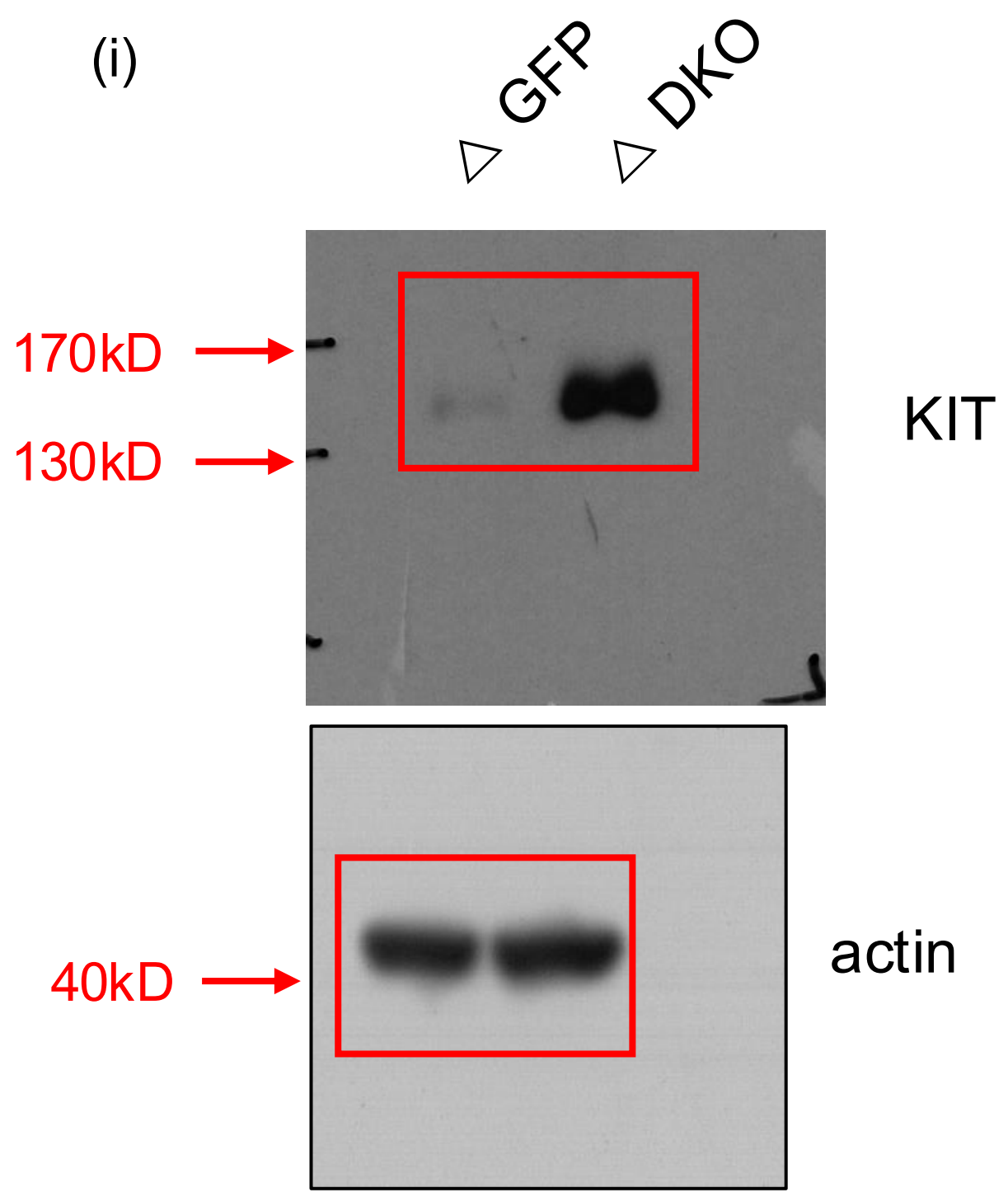

Figure 3

(b)

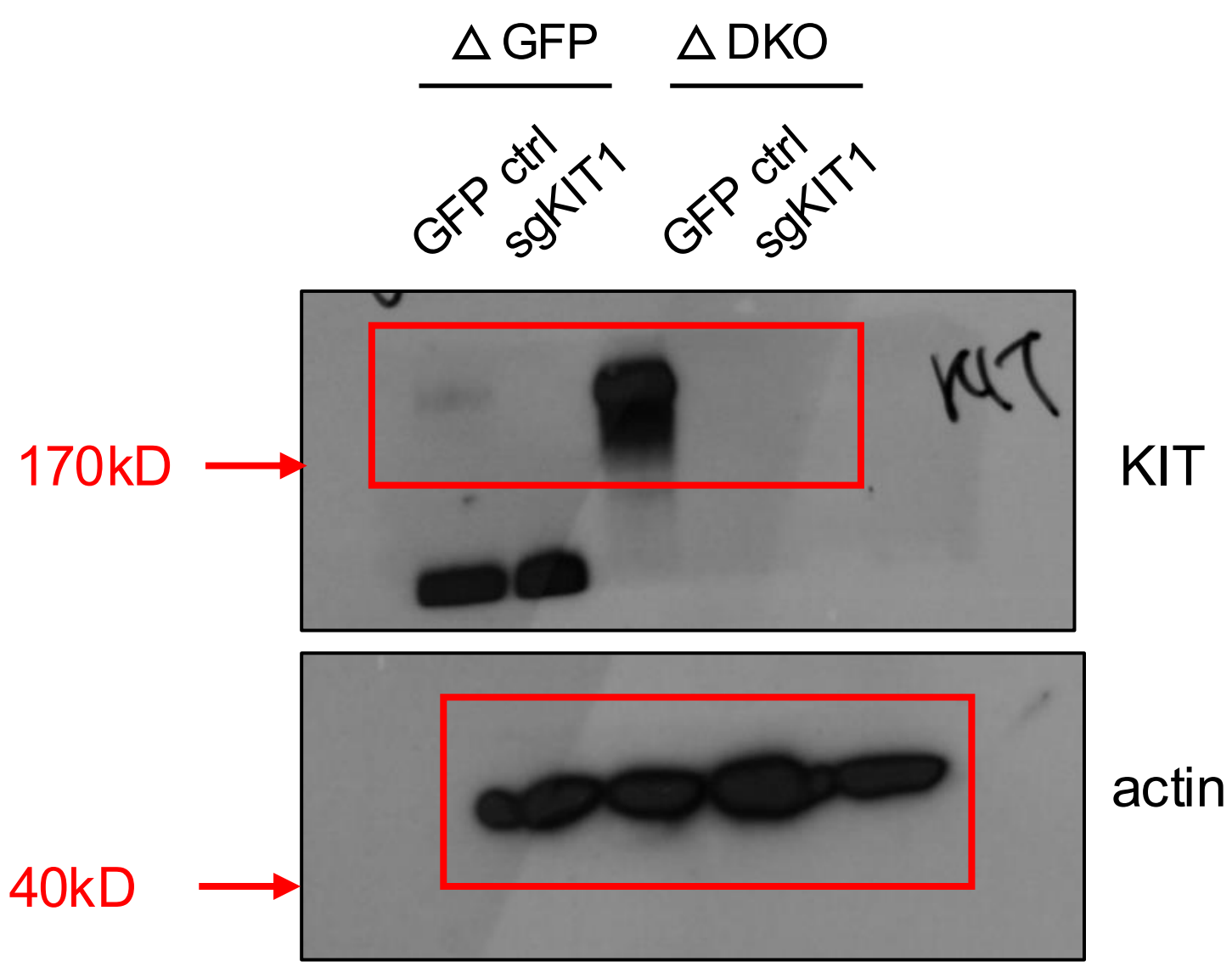
△GFP
△DKO
GFP ctrl
sgKIT1
GFP ctrl
sgKIT1
170kD
KIT
40kD
actin

(d)

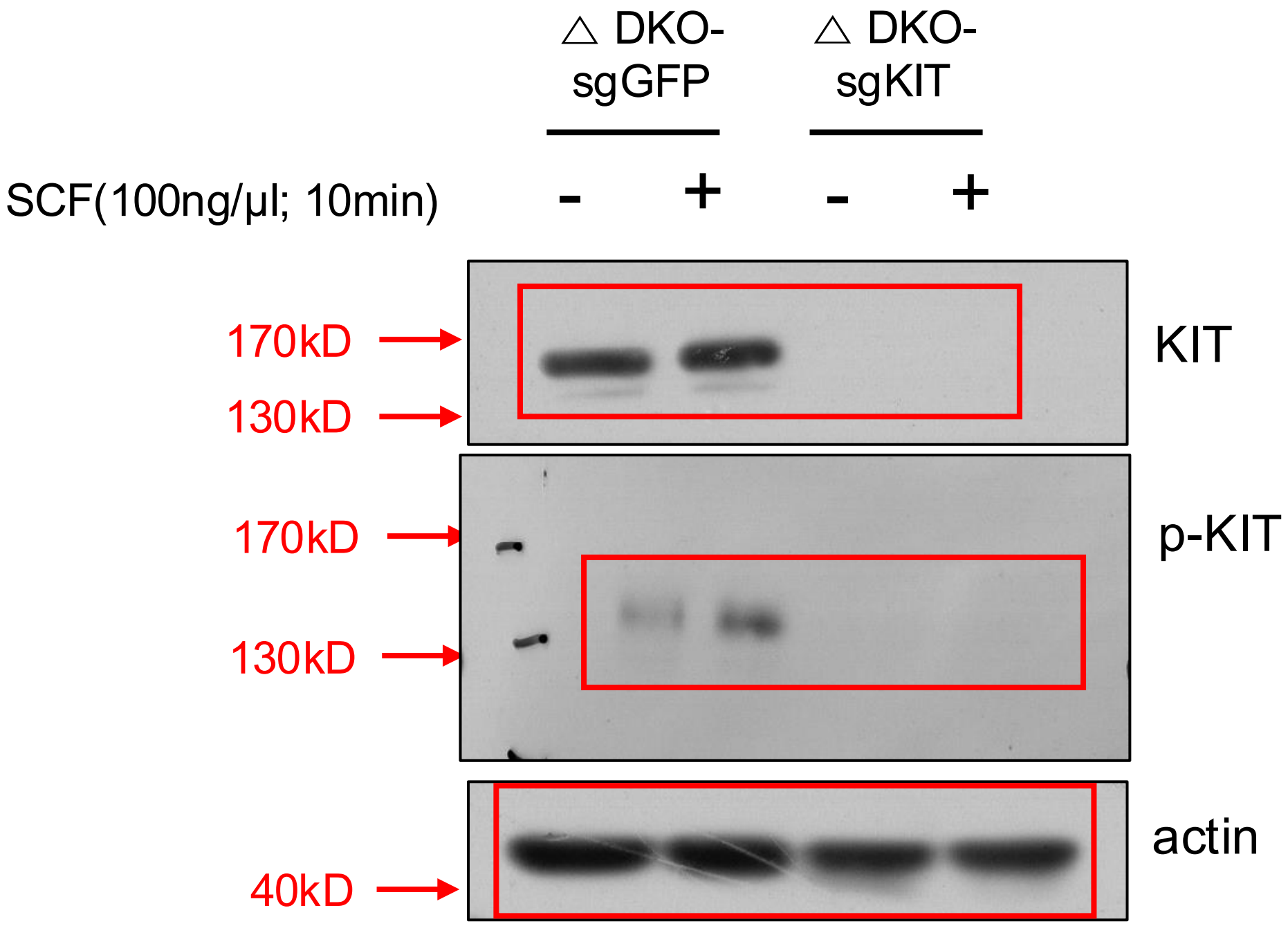
△ DKO-sgGFP
△ DKO-sgKIT
SCF(100ng/μl; 10min)
-
+
-
+
170kD
130kD
KIT
170kD
130kD
p-KIT
40kD
actin

Figure 4

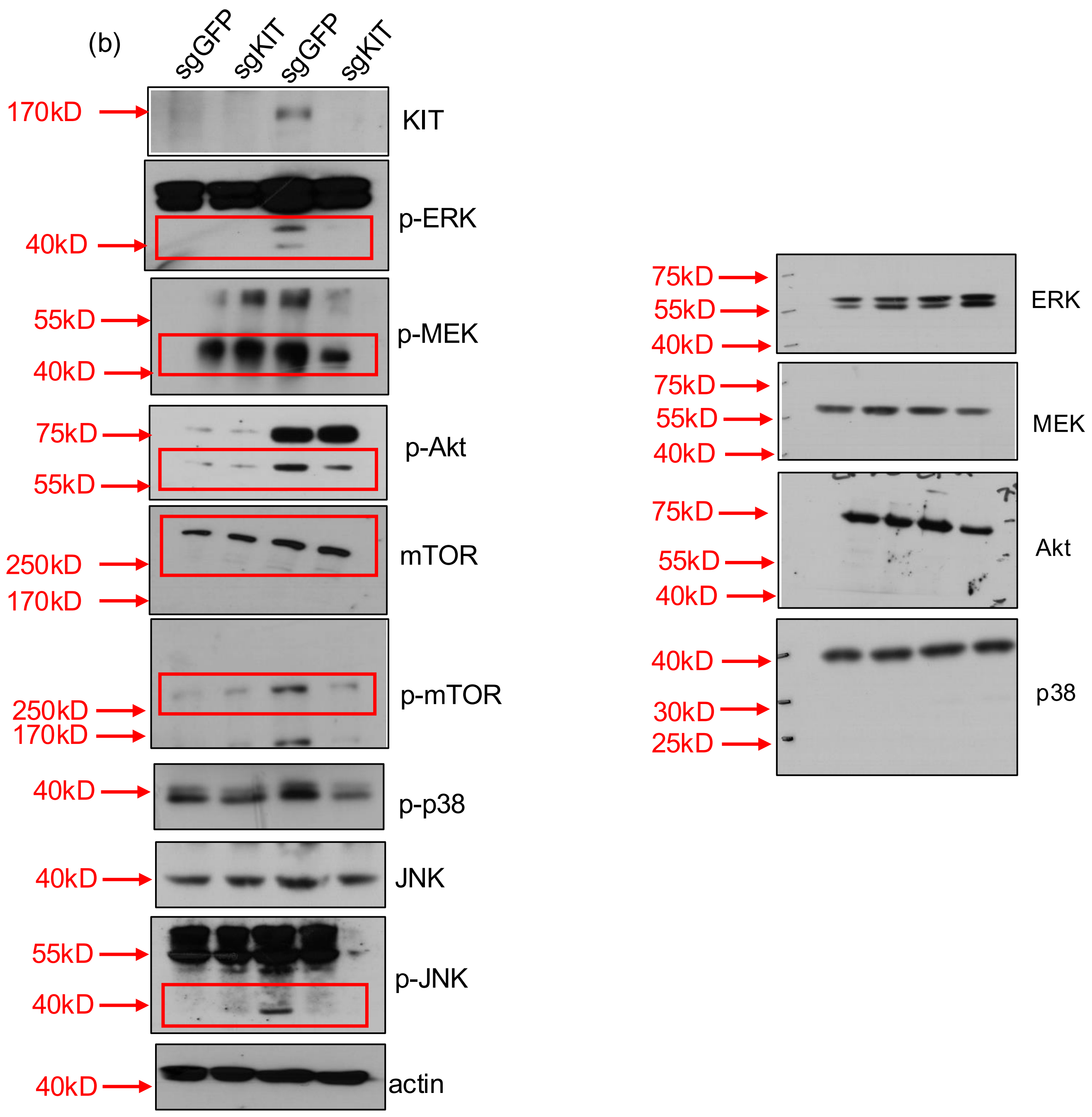
△ GFP
△ DKO
(b)
sgGFP
sgKIT
sgGFP
sgKIT
170kD
KIT
40kD
p-ERK
55kD
40kD
p-MEK
75kD
55kD
p-Akt
250kD
170kD
mTOR
250kD
170kD
p-mTOR
40kD
p-p38
40kD
JNK
55kD
40kD
p-JNK
40kD
actin
75kD
55kD
40kD
ERK
75kD
55kD
40kD
MEK
75kD
55kD
40kD
Akt
40kD
30kD
25kD
p38

Figure 5

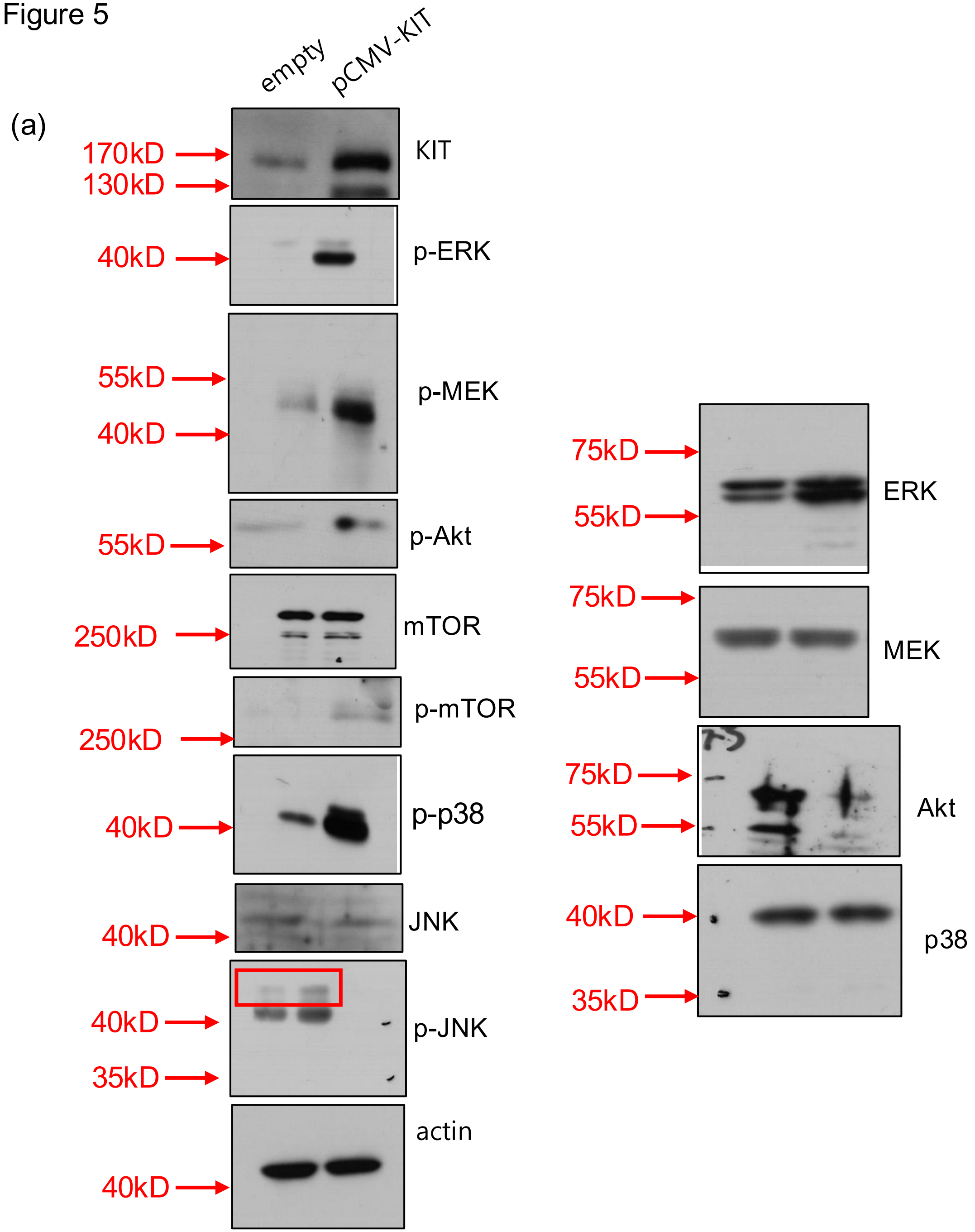

(a)
empty
pCMV-KIT
170kD
130kD
KIT
40kD
p-ERK
55kD
40kD
p-MEK
55kD
p-Akt
250kD
mTOR
250kD
p-mTOR
40kD
p-p38
40kD
JNK
40kD
35kD
p-JNK
40kD
actin
75kD
55kD
ERK
75kD
55kD
MEK
75kD
55kD
Akt
40kD
35kD
p38

Figure 6

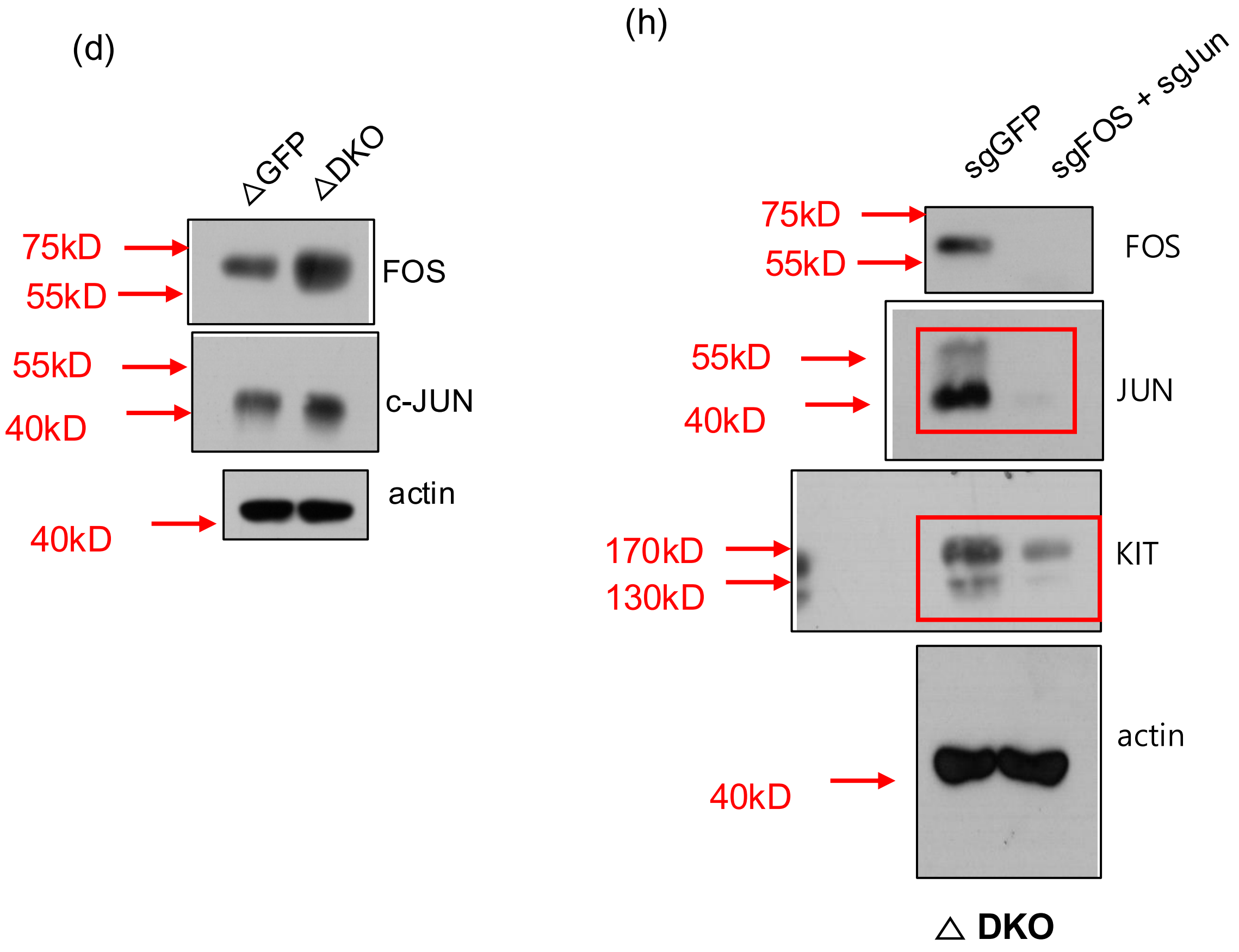
(d)
ΔGFP
ΔDKO
75kD
55kD
FOS
55kD
40kD
c-JUN
actin
40kD
(h)
sgGFP
sgFOS + sgJun
75kD
55kD
FOS
55kD
40kD
JUN
170kD
130kD
KIT
actin
40kD
△ DKO

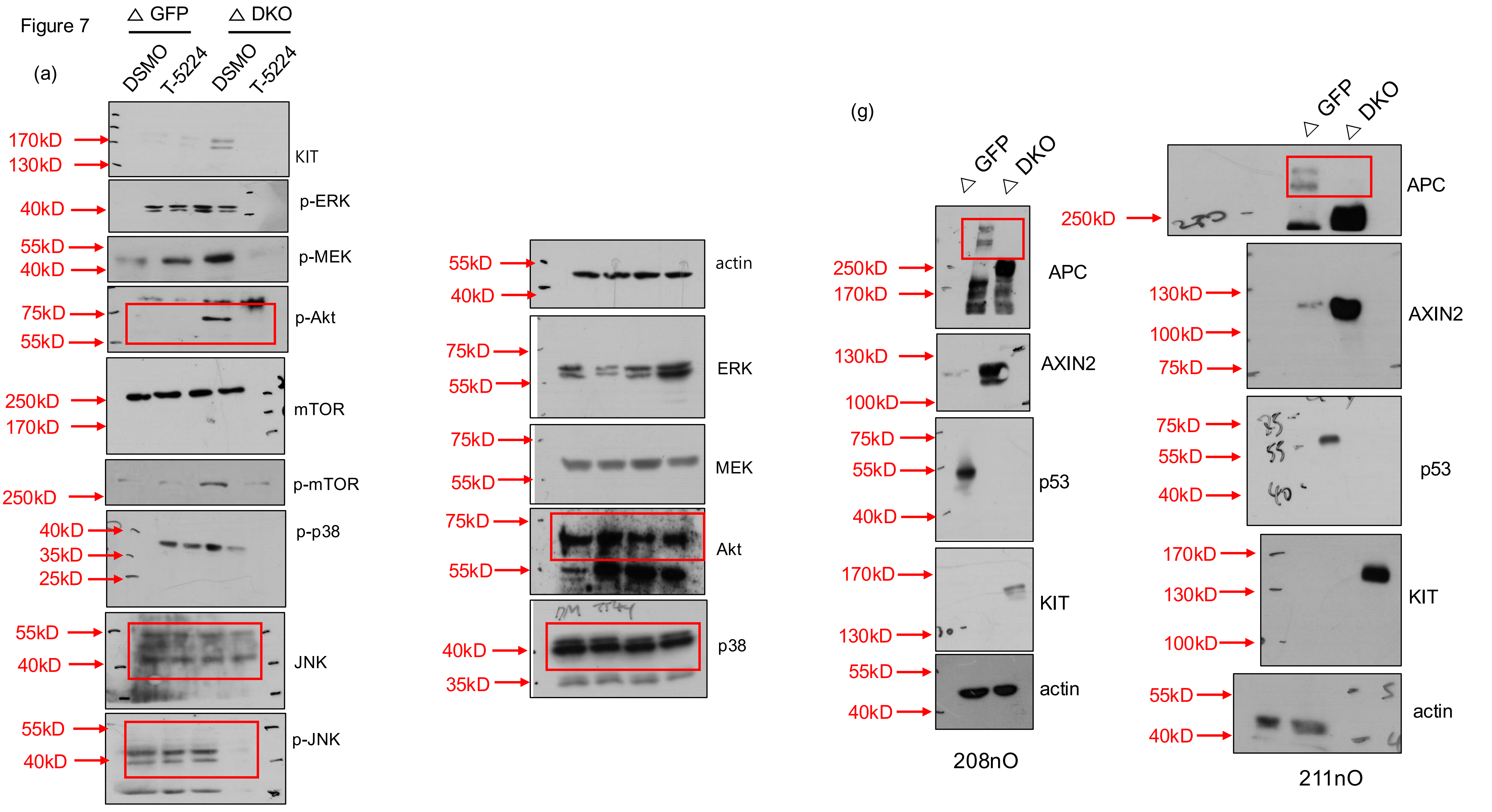
(a)
△ GFP
△ DKO
DSMO
T-5224
DSMO
T-5224
170kD
130kD
KIT
40kD
p-ERK
55kD
40kD
p-MEK
75kD
55kD
p-Akt
250kD
170kD
mTOR
250kD
p-mTOR
40kD
35kD
25kD
p-p38
55kD
40kD
JNK
55kD
40kD
p-JNK
55kD
40kD
actin
75kD
55kD
ERK
75kD
55kD
MEK
75kD
55kD
Akt
40kD
35kD
p38
(g)
△ GFP
△ DKO
250kD
170kD
APC
130kD
100kD
AXIN2
75kD
55kD
40kD
p53
170kD
130kD
KIT
55kD
40kD
actin
208nO
△ GFP
△ DKO
250kD
APC
130kD
100kD
75kD
AXIN2
75kD
55kD
40kD
p53
170kD
130kD
100kD
KIT
55kD
40kD
actin
211nO

Figure 7

Supplementary Figure S7

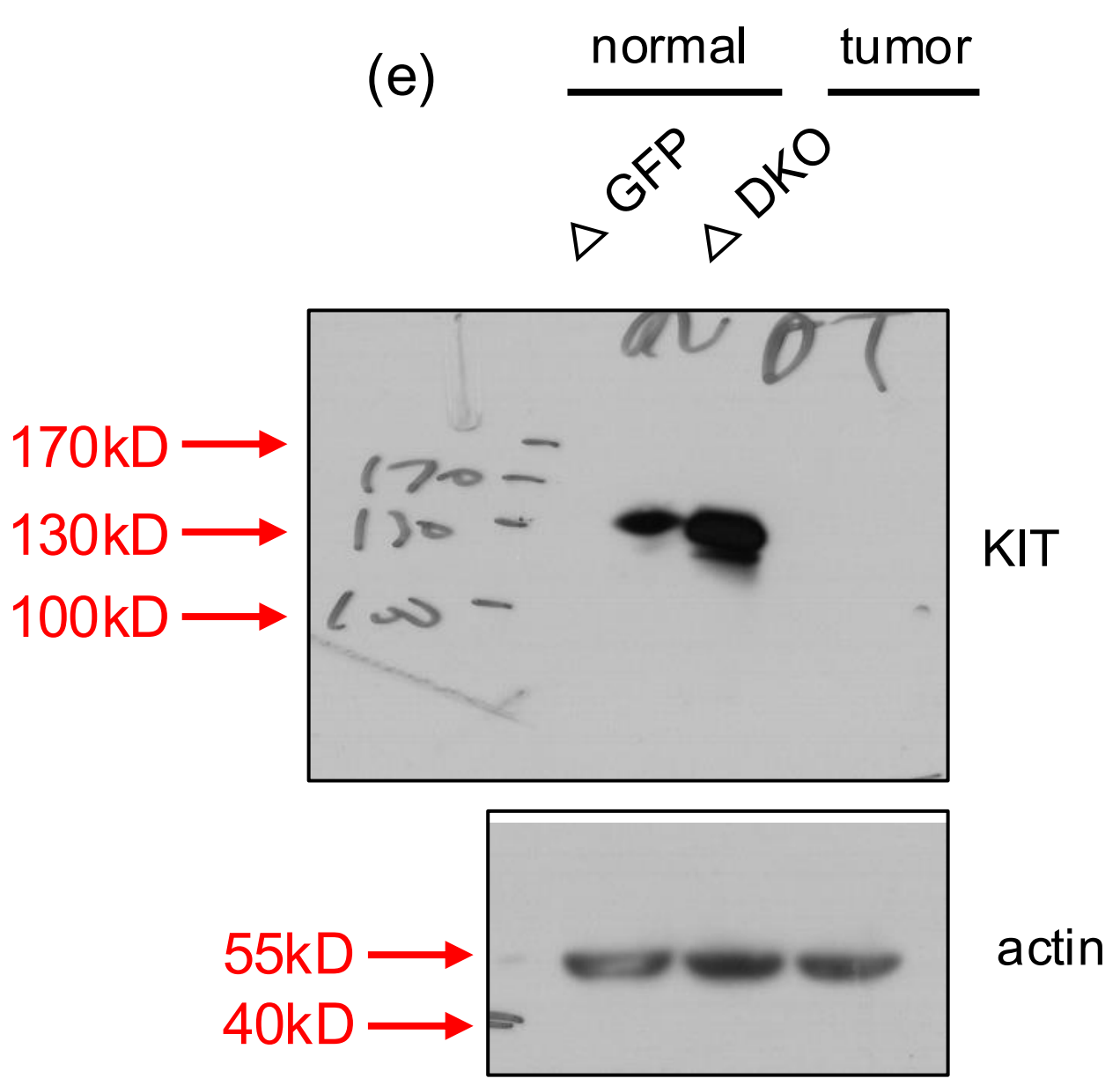

Supplement: Supplementary file 1 [file 41419_2026_9056_MOESM1_ESM.pdf]
