# Supplementary material for: Activation of KIT signaling promotes early tumorigenesis through the AP-1 pathway in APC/TP53 double-knockout human colon organoids
Source: Cell Death Dis. 2026 Jul 4;17(1):829. doi: 10.1038/s41419-026-09056-7 (PMC13612835; doi:10.1038/s41419-026-09056-7)
Supplement: Supplementary file 2 [file 41419_2026_9056_MOESM2_ESM.docx]

**Supplementary Figures S1-7.**

**Supplementary Figure S1.**

**Supplementary figure S1. Genetic landscape and validation of Wnt pathway activation in ΔDKO organoids**

**(a)** Oncoplot depicting genomic alterations in 423 colorectal cancer samples. Among 423 samples, ***APC* (71%) and *KRAS* (43%)** mutations were observed.

**(b)** Oncoplot depicting genomic alterations in 423 colorectal cancer samples. Among 423 samples, ***APC* (71%) and *SMAD4* (14%)** mutations were observed.

**(c)** Oncoprint showing the mutation profile of colorectal tumor tissue (OG203T). *APC* harbors a nonsense mutation, whereas *TP53* harbors a missense mutation. Colors indicate mutation types.

**(d)** ATP-based cell viability assay showing that cellular metabolic activity in WENR vs. EN conditions (n=3). Δ*APC* and ΔDKO organoids exhibited significantly higher viability in EN conditions, whereas ΔGFP and Δ*TP53* organoids showed reduced viability. Data are shown as mean ± SD; (*p < 0.05, ***p < 0.001).

**(e)** qRT-PCR for *AXIN2* in ΔGFP, Δ*APC*, Δ*TP53*, and ΔDKO organoids in the presence or absence of WNT/R-spondin. Expression normalized to 18s and horizontal bars represent the mean of n=3 individual experiments (*p < 0.05, **p < 0.01, ***p < 0.001).

**(f)** IHC images of representative ΔGFP and ΔDKO organoids stained for H&E and Ki-67. Scale bar = 200µm.

**(g)** Fluorescent EdU staining (red) in ΔGFP, Δ*APC*, Δ*TP53*, and ΔDKO organoids to assess proliferation. Nuclei are counterstained with DAPI (blue). Scale bar = 200 µm

**Supplementary Figure S2.**

**Supplementary figure S2.** **Integrated multi-omics analysis reveals transcriptional, chromatin, and epigenetic alterations in ΔDKO organoids.**

**(a, b)** Volcano plots (a) and summary table (b) of RNA-seq, ATAC-seq, H3K27ac-seq, and methyl-seq data (n=2) comparing ΔGFP and ΔDKO organoids. (a) Significant (FDR < 0.05) genes or regions are shown as non-black circles. Differentially regulated genes and regions are highlighted in red (upregulated/hyper-methylated) and blue (downregulated/hypo-methylated (b) Summary table showing the number of differentially expressed genes (RNA-seq), chromatin accessibility changes (ATAC-seq), H3K27ac enrichment changes, and DNA methylation differences between ΔGFP and ΔDKO organoids (|FC|) > 1.5 for RNA seq and |FC|) > 2 for ATAC-seq, H3K27ac-seq, methyl-seq)

**(c-e)** GO Biological Process enrichment analysis of ATAC-seq (c), H3K27ac-seq (d) and methyl-seq (e) datasets. For ATAC-seq and H3K27ac-seq, terms were derived from regions enriched or depleted in ΔDKO versus ΔGFP; for methyl-seq, terms were derived from hypo- or hyper-methylated regions. Enrichment analysis was performed using EnrichR (GO Biological Process 2025 library). Tumor-associated pathways are highlighted in red, and terms are ranked by enrichment. Asterisks indicate selected tumor-associated pathways that appear at lower ranks than those displayed. (c) Epithelial-to-mesenchymal transition (rank 25). (d) Negative regulation of vascular permeability (rank 9) and cell migration involved in sprouting angiogenesis (rank 15).

**(f)** Pie charts depicting the genomic annotation of all accessible regions in ΔGFP and ΔDKO organoids (left panel). Average signal intensity of k-means clustered ATAC-seq signals for differentially accessible regions that are exclusive to either ΔGFP organoids and ΔDKO organoids (right panel).

**(g)** Pie charts depicting the genomic annotation of all H3K27ac signals in ΔGFP and ΔDKO organoids (left panel). Average signal intensity of k-means clustered H3K27ac-seq signals for differentially accessible regions that are exclusive to either ΔGFP organoids and ΔDKO organoids (right panel).

**(h)** Scatterplot showing correlation of significant differential RNA-seq and ATAC-seq signals.

**(i)** Scatterplot showing correlation of significant differential RNA-seq and H3K27ac-seq signals.

**Supplementary Figure S3.**

**Supplementary figure 3. DNA methylation–associated transcriptional regulation in ΔDKO organoids**

**(a and b)** Methyl-seq analysis reveal key gene methylation changes in △DKO organoids. (a) Methyl-seq tracks around *LGR5* and *CA2* in △GFP and △DKO organoids. (b) qRT-PCR assay was performed to detect the expression of *LGR5* and *CA2* in △GFP and △DKO organoids. Expression normalized to 18s and horizontal bars represent the mean of n=3 individual experiments. (*p < 0.05, ***p < 0.001).

**(c)** qRT-PCR for *KIT* in ΔGFP and ΔDKO organoids in the presence or absence of WNT/R-spondin. Expression normalized to 18s and horizontal bars represent the mean of n=3 individual experiments (*p < 0.05, **p < 0.01).

**Supplementary Figure S4.**

**Supplementary figure S4** **Functional and transcriptomic consequences of KIT depletion in △DKO organoids.**

**(a)** IHC images of representative ΔDKO organoids with or without KIT, stained for H&E and Ki-67. Scale bar = 200µm.

**(b)** Fluorescent EdU staining (red) in ΔDKO organoids with or without KIT to assess proliferation. Nuclei are counterstained with DAPI (blue). Scale bar = 200 µm.

**(c)** Volcano plots of RNA-seq data comparing ΔDKO-sgGFP and ΔDKO-sg*KIT* organoids (n=2). Up-regulated genes are highlighted in red down-regulated genes are highlighted in blue (top). Summary table showing the number of differentially expressed genes in ΔDKO-sg*KIT* organoids relative to ΔDKO-sgGFP controls (bottom) (|FC| 2, FDR <0.05).

**(d, e)** Gene set functional enrichment analysis was performed using gProfiler2. Enrichment plots showing expression of ΔDKO-sgGFP significantly and positively correlated with (b)HALLMARK_G2M_CHECKPOINT, and (c)HALLMARK_E2F_TARGETS.

**(f, g)** Gene set functional enrichment analysis was performed using gProfiler2. Enrichment plots showing expression of ΔDKO-sg*KIT* significantly and positively correlated with (d) HALLMARK_APOPTOSIS, and (e) HALLMARK_P53_PATHWAY.

**Supplementary Figure S5.**

**Supplementary figure S5. Transcriptomic and functional effects of KIT overexpression in normal colon organoids**

**(a)** Volcano plots of RNA-seq data comparing ΔGFP-empty and ΔGFP-pCMV-KIT organoids (n=2). Up-regulated genes are highlighted in red down-regulated genes are highlighted in blue (top). Summary table showing the number of differentially expressed genes in ΔGFP-pCMV-KIT organoids relative to ΔGFP-empty organoids (bottom) (|FC| 2, FDR <0.05).

**(b, c)** Enrichment plots showing expression of ΔGFP-pCMV-KIT significantly and positively correlated with (g) HALLMARK_E2F_TARGETS, and (h) HALLMARK_G2M_CHECKPOINT.

**(d, e)** Enrichment plots showing expression of ΔGFP-empty significantly and positively correlated with (i) HALLMARK_ P53_PATHWAY, and (j) HALLMARK_OXYDATIVE_PHOSPHORYLATION.

**(f)** IHC images of representative ΔGFP organoids with empty or pCMV-*KIT* vector, stained for H&E and Ki-67. Scale bar = 300µm.

**(g)** Fluorescent EdU staining (red) in ΔGFP organoids with empty or pCMV-*KIT* vector to assess proliferation. Nuclei are counterstained with DAPI (blue). Scale bar = 200 µm.

**Supplementary Figure S6.**

**Supplementary figure S6. AP-1 binding and transcriptional regulation of KIT in ΔDKO organoids**

**(a)** qRT-PCR assay showing *KIT*, *FOS* and *JUN* expression in △GFP, △APC and △DKO organoids. Expression normalized to 18s and horizontal bars represent the mean of n=3 individual experiments (*p < 0.05, **p < 0.01, ***p < 0.001).

**(b)** Venn diagram of the number of peaks between ΔGFP and ΔDKO.

**(c)** Gene ontology biological process enrichment analysis of FOS ChIP-seq peaks identified top-ranked terms that were enriched in ΔDKO versus ΔGFP. Enrichment analysis was performed using EnrichR (GO Biological Process 2025 library) and terms are ranked by enrichment. Asterisks indicate selected tumor-associated pathways that appear at lower ranks than those displayed (Sprouting Angiogenesis, rank 21; right panel).

**(d)** IHC images of representative ΔDKO organoids with or without FOS and JUN, stained for H&E and Ki-67. Scale bar = 300µm.

**(e)** Fluorescent EdU staining (red) in ΔDKO organoids with or without FOS and JUN to assess proliferation. Nuclei are counterstained with DAPI (blue). Scale bar = 200 µm.

**Supplementary Figure S7.**

**Supplementary Figure S7.** **Pharmacological inhibition of AP-1 suppresses KIT signaling and tumorigenesis in ΔDKO Organoids**

**(a)** IHC images of representative ΔDKO organoids with DMSO or T-5224 treatment, stained for H&E and Ki-67. Scale bar = 300µm.

**(b)** Fluorescent EdU staining (red) in ΔGFP and ΔDKO organoids with DMSO or T-5224 treatment to assess proliferation. Nuclei are counterstained with DAPI (blue). Scale bar = 200 µm.

**(c)** Gene set functional enrichment analysis was performed using gProfiler2. Gene set enrichment analysis of 208 genes differentially expressed between △GFP and △DKO organoids (n = 2) identified altered Hallmark signaling pathways.

**(d)** qRT-PCR assay showing *KIT* expression in △GFP, △DKO organoids derived from normal tissue and in tumor organoids derived from the paired tumor tissue. Expression normalized to 18s and horizontal bars represent the mean of n=3 individual experiments (**p < 0.01, ***p < 0.001).

**(e)** Western blotting showing the KIT protein levels in △GFP, △DKO organoids derived from normal tissue and in tumor organoids derived from the paired tumor tissue.

**(f)** Box plot showing *KIT* expression in CRC samples (*n* = 275) and normal samples (*n* = 349) from The Cancer Genome Atlas (TCGA) databases. Red represents CRC samples and gray represents normal samples. *p < 0.05.

**Supplementary Figure S8.**

(Separate attachment)

**Supplementary figure S8. Raw blot related to Figures.**
